# Animal and vegetal materials of mouse oocytes segregate at first zygotic cleavage: a simple mechanism that makes the two-cell blastomeres differ reciprocally from the start

**DOI:** 10.1093/molehr/gaae045

**Published:** 2024-12-30

**Authors:** Thomas Nolte, Reza Halabian, Steffen Israel, Yutaka Suzuki, Roberto A Avelar, Daniel Palmer, Georg Fuellen, Wojciech Makalowski, Michele Boiani

**Affiliations:** Department of Cell and Tissue Dynamics, Max Planck Institute for Molecular Biomedicine, Münster, Germany; Institute of Bioinformatics, Faculty of Medicine, University of Münster, Münster, Germany; Department of Cell and Tissue Dynamics, Max Planck Institute for Molecular Biomedicine, Münster, Germany; Department of Computational Biology and Medical Sciences, Graduate School of Frontier Sciences, University of Tokyo, Chiba, Japan; Institute for Biostatistics and Informatics in Medicine and Ageing Research, Rostock University Medical Center, Rostock, Germany; Institute for Biostatistics and Informatics in Medicine and Ageing Research, Rostock University Medical Center, Rostock, Germany; Institute for Biostatistics and Informatics in Medicine and Ageing Research, Rostock University Medical Center, Rostock, Germany; Institute of Bioinformatics, Faculty of Medicine, University of Münster, Münster, Germany; Department of Cell and Tissue Dynamics, Max Planck Institute for Molecular Biomedicine, Münster, Germany

**Keywords:** animal model, blastomere, embryo development, fertilization, gene expression, ICSI, oocyte

## Abstract

Recent advances in embryology have shown that the sister blastomeres of two-cell mouse and human embryos differ reciprocally in potency. An open question is whether the blastomeres became different as opposed to originating as different. Here we wanted to test two relevant but conflicting models: one proposing that each blastomere contains both animal and vegetal materials in balanced proportions because the plane of first cleavage runs close to the animal–vegetal axis of the fertilized oocyte (meridional cleavage); and the other model proposing that each blastomere contains variable proportions of animal and vegetal materials because the plane of the first cleavage can vary – up to an equatorial orientation – depending on the topology of fertilization. Therefore, we imposed the fertilization site in three distinct regions of mouse oocytes (animal pole, vegetal pole, equator) via ICSI. After the first zygotic cleavage, the sister blastomeres were dissociated and subjected to single-cell transcriptome analysis, keeping track of the original pair associations. Non-supervised hierarchical clustering revealed that the frequency of correct pair matches varied with the fertilization site (vegetal pole > animal pole > equator), thereby, challenging the first model of balanced partitioning. However, the inter-blastomere differences had similar signatures of gene ontology across the three groups, thereby, also challenging the competing model of variable partitioning. These conflicting observations could be reconciled if animal and vegetal materials were partitioned at the first cleavage: an event considered improbable and possibly deleterious in mammals. We tested this occurrence by keeping the fertilized oocytes immobilized from the time of ICSI until the first cleavage. Image analysis revealed that cleavage took place preferentially along the short (i.e. equatorial) diameter of the oocyte, thereby partitioning the animal and vegetal materials into the two-cell blastomeres. Our results point to a simple mechanism by which the two sister blastomeres start out as different, rather than becoming different.

## Graphical Abstract

**Graphical Abstract:**
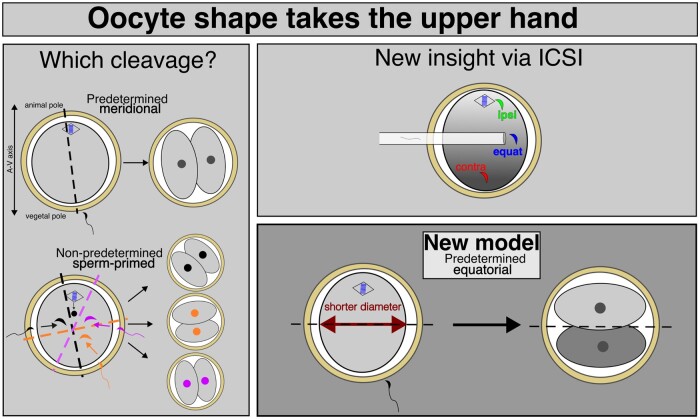
Current models disagree on the orientation of first zygotic division in relation to the sperm entry site. Through ICSI, we revealed that the oocyte shape prevails over the sperm entry site.

## Introduction

It is still unclear why the blastomeres of the two-cell mammalian embryo differ in cell potency from one another, as shown in mice (Piotrowska *et al.*, 2001; Casser *et al.*, 2017) and also reported more recently in humans (Junyent *et al.*, 2024). This question is intimately connected to the other question of how the fertilized oocyte (zygote) divides mitotically to form the two blastomeres. However, there is diversity of opinions even on such a basic cytological event. One prevalent model in mice posits that the first zygotic cleavage proceeds along the meridian that connects the oocyte’s animal (A) and vegetal (V) pole, irrespective of the sperm entry point. Oocyte polarity is not as plainly visible in mice and in most mammals as it is in other organisms, for example, *Xenopus laevis*, therefore, the A pole is assumed to be located where the second polar body (PB2) is extruded upon fertilization, and the diametrically opposite side is, by default, the V pole (Zernicka-Goetz, 1998; Tam, 2002; Simerly *et al.*, 2019). Following the mindset of the above model, cleavage would be predetermined, and each two-cell blastomere would contain both A and V materials in balanced proportions. A second, equally prevalent model posits that the first cleavage axis is not predetermined, instead, it is acquired after fertilization, being influenced by the apposition of the two pronuclei in the ooplasm, which, in turn, is influenced by the sperm entry point (Piotrowska and Zernicka-Goetz, 2001; Plusa *et al.*, 2002b; Hiiragi and Solter, 2004; Piotrowska-Nitsche and Chan, 2013). Following the mindset of the second model, each two-cell blastomere would contain variable proportions of A and V materials, up to the case of extremely different proportions when the cleavage is equatorial and the partition is in fact a segregation. Assuming that such materials have instructive properties, the consequences of the two models for the embryo are opposite; if the sister blastomeres received balanced proportions of A and V materials, then the blastomeres would originate as equal, whereas if the proportions received were unbalanced, then the sister blastomeres would be different from the start. This difference between models and which one is closer to reality is relevant to the conceptualization of early mouse development, which has many times, though not always, shown us the way in other mammalian species, including humans.

For context, we recapitulate what is known about the properties of two-cell stage blastomeres. Scholarly books on developmental biology have taught for more than 60 years that the two-cell stage blastomeres in mammals were as totipotent as the zygote, i.e. they both had the same ability to produce an entire individual (twins) after dissociation into single cells (Tarkowski, 1959). This notion was fostered by the historical studies of Hans Driesch performed in two-cell sea urchin embryos at the end of the 19th century (Driesch, 1964 (1892)). Each dissociated blastomere would develop into a pluteus larva, albeit not always of the same size, and this behaviour seemed to relate to how the plane of the first cleavage was oriented (reviewed in Gardner, 1996). Increasing evidence from 2017 onwards has been showing that when mouse blastomeres are separated at the two-cell stage, in most cases, only one blastomere is able to develop into a viable blastocyst or a live mouse (Casser *et al.*, 2017; Krawczyk *et al.*, 2021; Maemura *et al.*, 2021). This full developmental potential is probably attained on condition that the blastomere progeny accrues sufficient epiblast cells by the blastocyst stage (Morris *et al.*, 2012). In addition to these functional differences, molecular differences have also been identified between mouse blastomeres at the two-cell stage (Hupalowska *et al.*, 2018; Wang *et al.*, 2018, 2021). These findings prompted a reassessment of the notion that blastomeres of the same two-cell embryo have identical potency, i.e. ability for monozygotic twin formation in model and farm species, such as mouse, rabbit, sheep, and pig (Boiani *et al.*, 2019). Meanwhile, a study in humans has also shown that the properties of the two blastomeres are different and relate to the symmetric versus asymmetric division of their progeny cells at the 8- to 16-cell stage transition (Junyent *et al.*, 2024). Then again, how the differences originate at the two-cell stage remains largely unknown.

We hypothesize that the differences of potency between blastomeres of the same two-cell embryo (Casser *et al.*, 2017) are rooted, at least in part, in the orientation of the first zygotic cleavage. Our hypothesis builds on the rediscovery of the historical studies of Friedrich Seidel performed in rabbit zygotes between the early 1950s and the early 1960s. Seidel proposed that inter-blastomere differences at the two- and four-cell stages depend, at least in part, on the partition of oocyte territories through the orientation of the first cleavage after fertilization (Seidel, 1952, 1960). If the cleavage axis was meridional, i.e. along the A–V axis, then the two blastomeres should inherit equal proportions of A and V materials, and the cellular properties could be similar, if not identical, to each other; whereas if the cleavage axis was equatorial, i.e. perpendicular to the A–V axis, then the two blastomeres should inherit different proportions of A and V materials, and the cellular properties could be distinct. We use the conditional form because the molecular nature of such materials and the boundary between them is still elusive. Regarding the molecular nature, only a handful of gene candidates whose products are asymmetrically distributed have been named in mouse oocytes to date (Antczak and Van Blerkom, 1997, 1999; Evans *et al.*, 2000; Duncan *et al.*, 2005; Edwards, 2005; Schulz and Roberts, 2011). It is also possible that the A and V materials, rather than being gene products, are cellular structures, such as membrane- and non-membrane-bound organelles (Casser *et al.*, 2018). Concerning the boundary, the prevalent view is that it is perpendicular to the A–V axis, however, historical histochemical studies by Albert Dalcq suggested that the boundary could be parallel rather than perpendicular to the A–V axis in rat oocytes (Dalcq, 1957; Denker, 1983; Gardner, 1999). Either way, caution is recommended for the interpretation of histochemical results, because differences of signal intensity inside the specimen are influenced by the method of cell fixation (Littwin and Denker, 2011).

The purpose of the present investigation was to test the hypothesis that the orientation of the first zygotic cleavage contributes to the different properties of two-cell stage blastomeres in mice. Considering how confined the problem is (merely the division of one cell), how feasible it is to experimentally manipulate the early mammalian embryo, and the technical possibilities at our disposal today, it should be possible to understand the first zygotic cleavage in totality. Yet, so far, this has not been the case. This quest has been complicated by the variability in the sperm entry point and the reproducibility in assessing the cleavage patterns retrospectively, after the oocytes have been fertilized naturally or inseminated *in vitro*. Therefore, we started from the clean slate of metaphase II (MII) mouse oocytes, which allowed us to start before developmental processes were set in motion, and to prime the orientation of the first cleavage. The latter was achieved by imposing the fertilization at specifically predefined sites relative to the position of the MII spindle, via ICSI (Kimura and Yanagimachi, 1995). Accordingly, we succeeded in fertilizing the oocytes at the A pole, V pole or equator, and scoring their cleavage after they had been kept immobilized from the time of ICSI. Our innovative micromanipulation approach revealed that fertilized mouse oocytes divide in a way hitherto considered improbable and possibly even deleterious, i.e. along the equator, resulting in the separation of A and V materials in the blastomeres of the two-cell embryo. The implications of this cleavage behaviour are discussed in relation to the conceptualization of early embryonic development and the significance for human reproduction. Briefly, our results introduce the equatorial cleavage as a third path, in addition to the predetermined cleavage along the A–V meridian and the variable cleavage influenced by the sperm entry site. This third path entails the separation of A and V materials into the blastomeres, thereby pointing to a simple mechanism by which the sister blastomeres start out as different, rather than becoming different, consistent with the two-cell embryo properties reported in mice (Casser *et al.*, 2017) and humans (Junyent *et al.*, 2024).

## Materials and methods

### Compliance with regulations on animal experiments

All mice used were maintained in individually ventilated cages in the animal facility of the MPI Münster, with a controlled temperature of 22°C, a 14/10 h light/dark photoperiod and free access to water and food (Teklad 2020SX, ENVIGO RMS GmbH, Düsseldorf, Germany). Procedures used in this study followed the ethical guidelines of the FELASA (Federation of the European Laboratory Animal Science Associations) and the ARRIVE (Animal Research: Reporting of *In Vivo* Experiments) reporting guidelines. On the local regulatory level, mice were used for experiments according to the ethical approval issued by the Landesamt für Natur, Umwelt und Verbraucherschutz of the state of North Rhine-Westphalia, Germany (Permit number 81-02.04.2020.A405, ‘Die Zuteilung der zygotischen Totipotenz in den ersten Blastomeren: Eine Untersuchung der Ursprünge und Mechanismen im Mausmodell’).

### Collection of oocytes

Six- to eight-week-old B6C3F1 females were primed with 10 I.U. each of pregnant mare serum gonadotropin (PMSG; Pregmagon, Ceva Tiergesundheit, Düsseldorf, Germany) and hCG (Ovogest, MSD Tiergesundheit, Unterschleißheim, Germany) injected intraperitoneally 48 h apart at 5 p.m., and then killed by cervical dislocation to collect MII oocytes from oviducts. Cumulus cells were removed in hyaluronidase (CAT No. 151271, ICN Biomedicals, Costa Mesa, CA, USA) 50 I.U./ml in HEPES-buffered CZB medium (HCZB; Cavaleri *et al.*, 2006). HCZB contained polyvinylpyrrolidone (PVP, 40 kDa; CAT No. 529504, Calbiochem, EMD Biosciences, La Jolla, CA, USA) 0.2% w/v in place of bovine serum albumin (BSA). After removing the cumulus cells, MII oocytes were kept in 500 µl of α-MEM medium (CAT No. M4526, Sigma-Aldrich Chemie GmbH, Taufkirchen, Germany) containing 0.2% w/v BSA (Probumin, Milllipore, Kankakee, IL, USA) and 50 µg/ml gentamicin, in a Nunc 4-well plate (CAT No. 176740, Thermo Scientific, Bremen, Germany) without oil overlay at 37°C under 6% CO_2_ in air, until ICSI.

### Collection of sperm and cryopreservation

Caudae epididymis from two three-month-old CD1 male mice was collected, and spermatozoa were allowed to swim up in ∼1 ml Whittingham medium (Fraser and Drury, 1975) supplemented with 3% w/v BSA (CAT No. A3311, Sigma-Aldrich Chemie GmbH). Swim-up took place across a distance of ∼1.5 cm for 30 min under a humidified atmosphere of 6% CO_2_ in air at 37°C. The upper layer (∼200 μl) of swim-up medium was collected and incubated under pre-equilibrated mineral oil (CAT No. M8410, Sigma-Aldrich Chemie GmbH), to allow for sperm capacitation. After 1 h, the 200 μl drop of medium containing the spermatozoa was centrifuged (200 *g* for 20 min at room temperature) and then resuspended in half the volume of Whittingham medium. Aliquots of 5 µl of sperm suspension were placed at −80°C for 1 week and then stored in liquid nitrogen, until use for ICSI.

### ICSI and embryo culture

The ICSI was conducted on the stage of a Nikon TE2000-U microscope fitted with Nomarski optics, a Narishige micromanipulator (Model NT-88NE) and a piezo drill (PrimeTech Ltd, Ibaraki, Japan). The sperm heads were microinjected into the oocytes using a blunt-end glass needle (inner diameter 6–7 µm, outer diameter 8–9 µm) filled with 2–3 μl mercury at the tip. Injection volumes were controlled using a Gilmont GS-1200 micrometer syringe operated manually. At 14–15 h post-hCG, the MII oocytes were held in HCZB medium during the ICSI, following our micromanipulation protocol, in which the holding pipette is on the right-hand side and the ICSI pipette comes from the left, except that this time, the oocytes were also rotated to consistently inject the sperm head at 3 o’clock with the MII spindle at 9 o’clock (V pole ICSI), 3 o’clock (A pole ICSI) or 12 o’clock (equatorial ICSI). The oocytes were also immobilized when applicable. To this end, a slight but constant suction was applied consistently with the holding pipette. We kept the oocytes immobilized on the stage of the micromanipulator for 24 h to assess the relationship between the site of the ICSI and the first zygotic cleavage. We found empirically that cleavage was supported on the plastic bottom (not glass) under air, by adding 25% (v/v) of potassium (K) simplex optimization medium containing amino acids (KSOM(aa)) to the HCZB medium and preserving osmolarity via oil overlay, at a temperature of 37°C maintained via a Thermo Plate (TOKAI HIT Co. Ltd, Shizuoka-ken 418-0074, Japan). The KSOM(aa) medium was prepared in-house as per the original recipe (Summers *et al*., 2000) and contained both essential and non-essential free amino acids, 0.2% (w/v) BSA (Probumin, Millipore) and gentamicin (50 µg/ml). Once the relationship between the site of the ICSI and the first zygotic cleavage was determined, the injected oocytes in subsequent experiments were transferred into 500 µl of KSOM(aa) medium in a Nunc 4-well plate (CAT No. 176740, Thermo Scientific), without oil overlay at 37°C under 6% CO_2_ in air, until bisection on the next day.

### Bisection of two-cell embryos

Two-cell embryos arising in the time frame from 20 to 22 h post-ICSI were removed from the culture to perform bisection from 22 to 24 h post-ICSI. These embryos were transferred in groups of 12 to a micromanipulation drop on the stage of a Nikon Eclipse TE2000-U inverted microscope fitted with Nomarski optics, and holding and bisection needles in place. The bisection medium consisted of 0.2 mM D(+) glucose, 0.2 mM pyruvate, 10 mM lactate, 0.5% w/v BSA (CAT No. A3311, Sigma-Aldrich Chemie GmbH), in 0.9% w/v sodium chloride, as described previously (Casser *et al.*, 2017). We also made the bisection medium slightly hypertonic by using 95% of the water volume. The bisection tool was a TransferTip (ES) needle operated by a CellTram Vario (Eppendorf SE, 22339 Hamburg, Germany). The two-cell embryo was rotated using the holding and bisection needle to align the cleavage plane with the common axis of the two pipettes. The two-cell embryo was firmly held in place with the holding pipette by applying negative pressure (suction). The TransferTip was used to initially make a slit in the ZP at one pole and then to press the ZP gently at the equatorial region, causing one blastomere to be squeezed out. Suction in the holding pipette was then reduced and the other blastomere came out by performing the same procedure, except that the needle was pressed against the ZP below the equatorial region. When all 12 embryos had been bisected, which normally took 8–10 min, the pairs of blastomeres were collected and transferred to another medium/vessel using a bent (≈60° angle), mouth-operated pipette with a flame-polished tip. The individual blastomeres were either lysed in RNA buffer or allocated individually to culture in 75 µl KSOM(aa) medium in a 96-well plate with round bottoms (CAT No. 163320, Thermo Scientific), without oil overlay, at 37°C under 6% CO_2_ in air. Culture under these conditions took place for an additional 72 h until the blastocyst stage.

### Embryo transfer and postimplantation development

Blastocysts were transferred as pools of eight to one uterine horn of pseudo-pregnant CD1 recipients that had the copulation plug from vasectomized CD1 males 3 days prior to the embryo transfer. Typically, the recipients weighed between 25 and 30 g and were older than six weeks but no older than three months. Prior to surgery, CD1 recipients were anesthetized with ketamine (80 mg/kg)/Xylazine (16 mg/kg) combined with Tramadol (15 mg/kg) as pain therapy, dissolved in phosphate-buffered saline and delivered intraperitoneally to the mouse at 10 μl/g body weight. Embryos were delivered to the uterine lumen using a mouth-operated glass pipette through a hole made in the cranial region of the uterine horn using a 27G needle. Wounds in the skin were closed with metal clips (type Michel 7.5 × 1.75 mm). The surgery *per se* typically took 10–15 min per mouse. The post-surgery recovery area was warmed to 30°C using infrared lamps. After returning the animals into their holding cages, the pain therapy continued for three days via Tramadol in the drinking water (0.5 mg/ml). Pregnancies were recorded by cesarean section shortly prior to the natural term (embryonic day (E) 18.5).

### *In vitro* model of implantation (outgrowths)

We transferred twin blastocysts (72 h after two-cell embryo bisection) individually onto a feeder layer of γ-ray-inactivated (30 Gray) mouse embryonic fibroblasts (C3H background) grown to confluence in 96-well plates (flat bottom) previously, using our adaptation (Boiani *et al.*, 2002) of the outgrowth method (Armant *et al.*, 1986). The medium consisted of high-glucose Dulbecco’s modified eagle medium with high glucose (CAT No. D5671, Sigma-Aldrich Chemie GmbH) supplemented with 15% heat-inactivated foetal bovine serum (BioWest, Nuaillé, France), GlutaMAX 1× (CAT. No. 35050-038, Gibco at Thermo Scientific), penicillin/streptomycin 1× (CAT. No. P4333, Sigma-Aldrich Chemie GmbH), non-essential amino acids 1× (CAT. No. M7145, Sigma-Aldrich Chemie GmbH), mercaptoethanol 0.1 mM (CAT No. 31350-010, Gibco at Thermo Scientific) and 1000 Units/ml LIF (produced in-house).

### Transcriptome analysis of single blastomeres and single blastocysts

Single blastomeres or single blastocysts were lysed in 10× Lysis Buffer (CAT No. 635013, TaKaRa Bio Inc., Shiga, Japan), deposited in two 384-well plates (Mosquito^®^), for a total of 150 pairs of blastomeres (300 cells) and 78 blastocysts (36 intact, 42 twins from 21 pairs). The RNA was then extracted and converted to cDNA using a SMART-Seq Single Cell Kit (CAT No. 634470, TaKaRa Bio Inc.). Sequencing libraries were prepared using the Illumina Nextera XT DNA Library Preparation Kit (CAT No. FC-131-1024, Illumina Japan, Minato-ku, Tokyo, Japan). Libraries were sequenced on an Illumina NovaSeq 6000 platform to obtain two datasets of 4.2 ± 1.5 million total mapped reads per library for blastomeres (dataset 1 with 150-base paired-end reads and dataset 2 with 100-base single-end reads). The datasets for blastocysts had 2.9 ± 1.1 million total mapped reads per library (only 100-base single-end reads). In order to mitigate any potential bias arising from discrepancies between these libraries, only the initial reads from the paired-end dataset were preserved, while the last 50 bp of each read were trimmed. Consequently, the subsequent blastomeres analysis exclusively employed sequencing data from both libraries, wherein the preserved first reads from the paired-end dataset were adjusted to a standardized length of 100 bp.

Trimmomatic V.039 (https://doi.org/10.1093%2Fbioinformatics%2Fbtu170) was applied to trim reads and remove adapters with the following parameters; ‘SE ILLUMINACLIP:NexteraPE-PE.fa:2:30:10 LEADING:3 TRAILING:3 SLIDINGWINDOW:4:15 MINLEN:50’. A pre-built mouse reference set was downloaded from the 10× genomics repository (https://cf10xgenomics.com/supp/cell-exp/refdata-gex-mm10-2020-A.tar.gz) on 11 December 2022, which included the reference genome (GRCm38) and gene annotation (M23, Ensembl 98). In order to quantify the gene expression, the reads originated from blastomeres and blastocysts were separately mapped to the mouse reference genome using the STAR pipeline (https://doi.org/10.1093/bioinformatics/bts635) with the following options; ‘—soloType SmartSeq—readFilesManifest manifest.tsv—soloUMIdedup Exact NoDedup—soloStrand Unstranded—genomeDir path_to/enome_dir/—soloFeatures Gene SJ—outSAMtype BAM SortedByCoordinate—quantMode GeneCounts TranscriptomeSAM—outTmpKeep All’. A total of 124 (62 pairs) of the starting 300 cells (150 blastomere pairs) were retained. A total of 69 of the starting 78 blastocysts were retained.

The resultant raw count matrix derived from blastomeres, composed of 124 cells (62 twin blastomeres and 32 285 genes), was processed using the SCANPY toolkit (Wolf *et al.*, 2018). This phase involved generating quality control metrics and eliminating cells and genes of poor quality. During this step, cells exhibiting fewer than 9500 detected genes and mitochondria gene counts exceeding 0.02% were excluded from subsequent analyses. Due to this filtering process, the number of cells decreased from 124 to 117, resulting in the loss of corresponding mates for some twin blastomeres. To address this, the unpaired cells were removed, leaving us with a final set of 110 cells (55 pairs) for further analysis. Those genes that were expressed in less than two cells and had fewer than 10 reads mapped to them were subsequently filtered out. These filtering steps reduced the initial 32 285 genes to 21 778. Afterwards, each gene’s transcript level was rendered as transcripts per million (TPM) using an in-house Python script. The length of each gene was obtained utilizing the GenomicFeatures package (Lawrence *et al.*, 2013) to accomplish this. Following that, the median TPM value was calculated for each gene across 110 blastomeres. The genes were then sorted in descending order based on their median TPM values. In a subsequent step, genes annotated as ‘predicted genes’ were removed from the dataset, and only the genes with an official symbol were retained. Finally, only those genes with a non-zero median TPM were retained, resulting in a total of 7492 genes for further analysis. The filtering steps are summarized in Supplementary Table S1.

The analysis of the count matrix corresponding to single blastocysts (dataset 2) followed the blastomeres procedure, with a few distinctions in the specimen-filtering criteria. Specimens characterized by fewer than 4000 detected genes were excluded, and, unlike blastomeres, no filtering was applied based on the mitochondrial genes detected due to their normal counts. The subsequent steps in blastocyst processing, including TPM calculation, mirrored those applied to blastomeres as well. Consequently, the processed blastocysts encompassed a total of 69 specimens, comprising 38 twin blastocysts (19 pairs) and 31 intact blastocysts. The latter were defined as those in which the two-cell embryo was not bisected (unlike the twins). This dataset, comprising 7660 genes, was subjected to further analysis. The filtering steps are summarized in Supplementary Table S1.

### Functional enrichment analysis

Functional enrichment analysis was performed using *Webgestalt* (Zhang *et al.*, 2005; Elizarraras *et al.*, 2024) at https://www.webgestalt.org and *Enrichr* (Chen *et al.*, 2013) at https://maayanlab.cloud/Enrichr/. We selected the GOs ‘Biological Process’ and ‘Cellular Component’. Terms with a FDR ≤ 0.05 were considered enriched.

### Analysis of published time-lapse videos of the first zygotic cleavage

The time-lapse videos were downloaded from the supplementary data of the respective papers (Cooke *et al.*, 2003; Gray *et al.*, 2004; Hiiragi and Solter, 2004; Motosugi *et al.*, 2005; Plusa *et al.*, 2005; Yamagata *et al.*, 2009; Wong *et al.*, 2010; Mizutani *et al.*, 2012; Athayde Wirka *et al.*, 2014; Yang *et al.*, 2015, 2024; Iwata and Mio, 2016; Ebner *et al.*, 2017; Yao *et al.*, 2018, 2022; Desai and Gill, 2019; Mashiko *et al.*, 2020; van Marion *et al.*, 2021; Baran and Pisko, 2022; Currie *et al.*, 2022; Jin *et al.*, 2022; Okabe *et al.*, 2023; Suzuki *et al.*, 2023; Zhao *et al.*, 2024). The videos were played, and two screenshots were taken shortly after extrusion of the PB2 and immediately after the first cleavage. Both screenshots were then imported to Fiji Image-J (Schindelin *et al.*, 2012). One line was drawn through the A–V axis of the zygote (identified using the PB2) and a second line was drawn through the plane of the first cleavage. The angle between the two axes was then measured. The same procedure was applied to measure the angle of first cleavage relative to the shorter diameter of the zygote.

### Data and statistical analysis

The values presented in the figures are mean values ± SDs. Statistics was performed using the Wilcoxon test or chi-squared test with JMP Pro software v.16 (SAS Institute GmbH, 69118 Heidelberg, Germany). Histograms and statistical distributions were generated in GraphPad Prism software.

## Results

### A robust method of fertilization that is suitable for testing the relationship between the orientation of zygotic cleavage and properties of two-cell stage embryos and their blastomeres

The glitch with current models of the first zygotic cleavage is that they are difficult to test empirically. This is because: (1) oocytes fertilized *in vivo* or inseminated *in vitro*, in which the starting point of analysis is post-fertilization, are the most commonly used material, whereby the relevant processes are already in progress; (2) the spermatozoon has a wide region of the oocyte surface where it can enter, and the specific entry site is almost random; and (3) the cytological landmark used to orient the fertilized oocytes is the PB2, which can move away from its original position. Therefore, we took as our starting point mouse MII oocytes, profiting from the fact that fertilization has yet to occur (hence we could still impose it at specific sites), the developmental processes have yet to start, and the meiotic MII spindle defines the A pole unambiguously.

**Figure 1.**
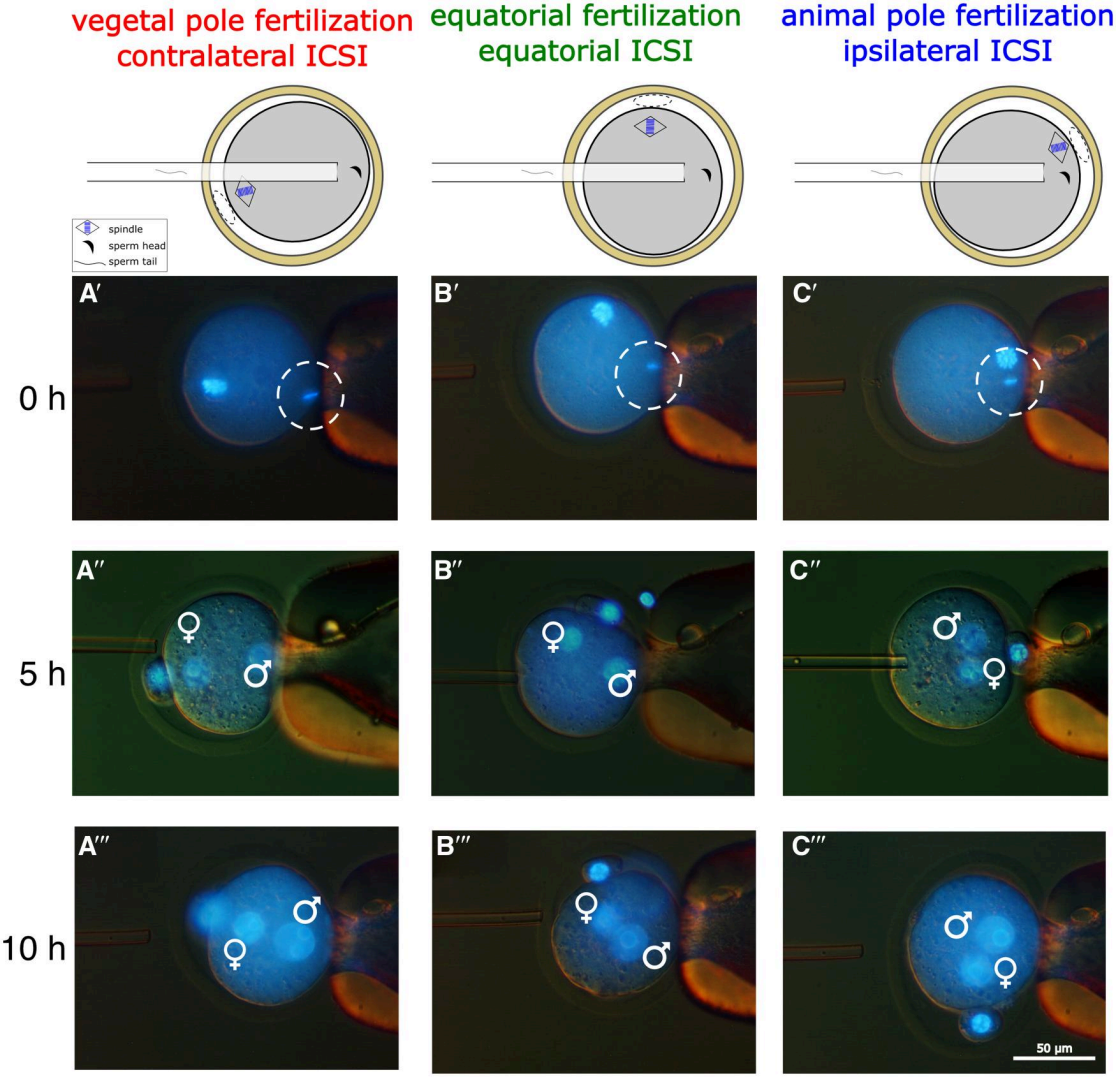
**Sperm deposition at specifically predefined sites of the mouse ooplasm relative to the position of the MII spindle**. (**A–C**) MII mouse oocytes (B6C3F1) at 14–15 h post-hCG were held in position by gentle suction of the zona pellucida with a holding pipette at the 3 o’clock position and subcortically injected with a single sperm head (CD1) using a piezo-driven needle approaching from the 9 o’clock position. (**A′–C′**) A single sperm head (encircled) was deposited in the cortical ooplasm opposite the spindle, i.e. contralateral (vegetal pole), half-way between poles (equator), or next to the spindle, i.e. ipsilateral (animal pole) of MII oocytes preloaded with Hoechst 33342. (**A″–C″**) Two pronuclei (♂♀) had formed 5 h after ICSI, attesting that the activation of the oocytes had been successful. (**A″′–C″′**) Pronuclei had enlarged and moved further toward the centre of the oocyte 10 h after ICSI. Blue colour in photographs is from DNA staining with Hoechst 33342 (1 µg/ml). MII, metaphase II.

Using a micromanipulator fitted with Nomarski optics, we oriented MII oocytes so as to have the MII meiotic spindle (A pole marker) placed at 3, 9, or 12 o’clock. A single sperm head was introduced from 9 o’clock and released subcortically at 3 o’clock (Fig. 1A–C). We used the same batch of spermatozoa, which were aliquoted and cryopreserved for the purpose, throughout the study for more consistency. A small sample of oocytes were stained with Hoechst 33342 for visualization purposes to confirm the spatial relationship between sperm deposition site and MII spindle, that is, the fertilization topology (Fig. 1A′–C′). As a result of the three ICSI sites, the oocytes became fertilized ipsilaterally (A pole), contralaterally (V pole) or half-way between (equatorially), as seen from the standpoint of the MII spindle. Immediate oocyte survival rates after the ICSI were similar across the three groups: ipsilateral 81 ± 8% (566 injections in 14 ICSI sessions), contralateral 85 ± 9% (608 injections in 14 sessions), and equatorial 87 ± 6% (626 injections in 14 sessions). When removed from the micromanipulator, cultured in an incubator, and then briefly returned to the micromanipulator for imaging at 5 and 10 h post-ICSI, the fertilized oocytes had invariably formed two pronuclei (5 h, Fig. 1A″–C″; 10 h, Fig. 1A″′–C″′), notwithstanding a concern that the A pole should be avoided in the ICSI (see the Discussion).

**Figure 2.**
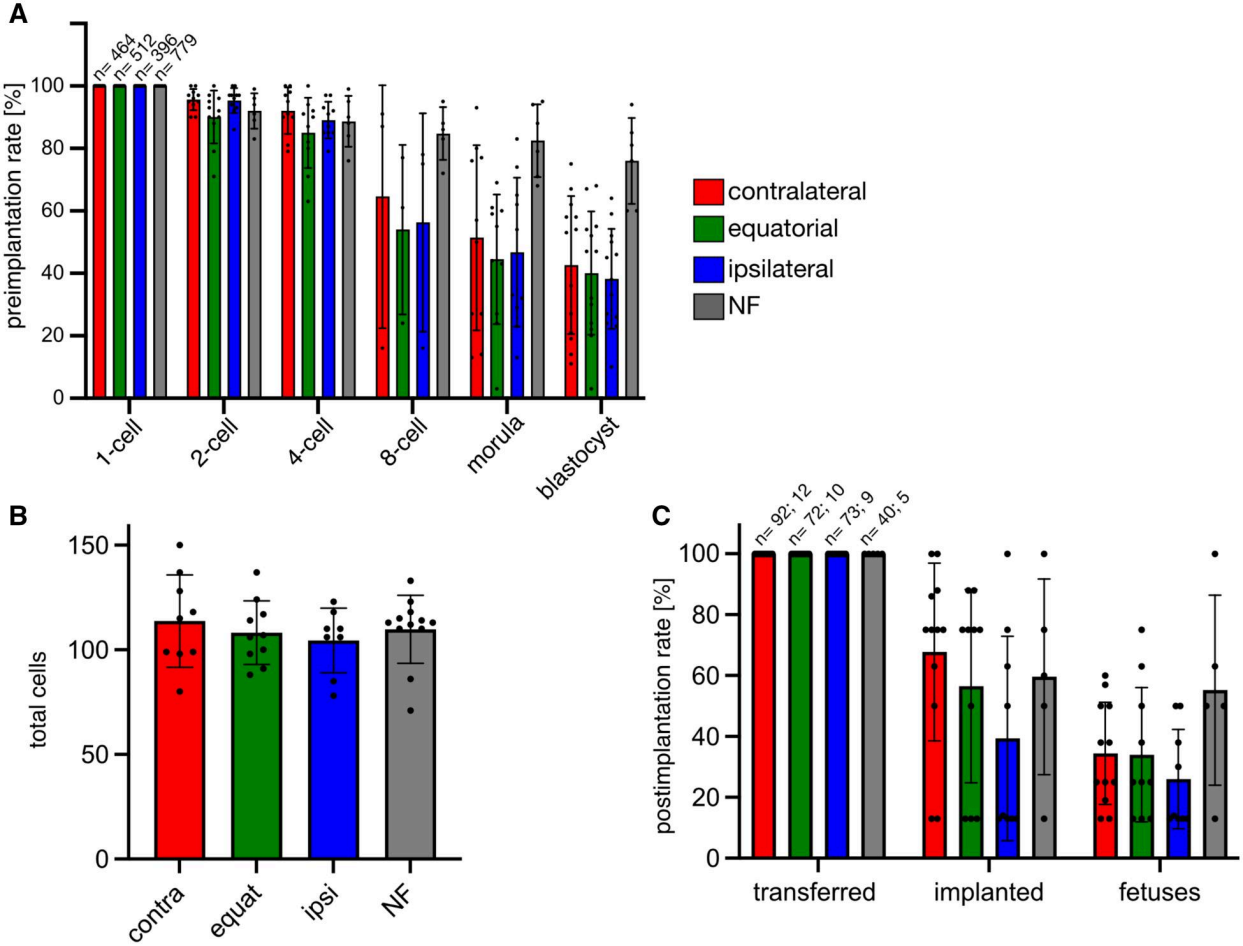
**Full development after site-specific ICSI**. (**A**) Blastocysts formed at rates not significantly different from each other in all three ICSI groups (contralateral 43 ± 22%, equatorial 40 ± 20%; ipsilateral 38 ± 16%; *P* > 0.555; Wilcoxon test), albeit lower than those of oocytes subjected to natural fertilization (NF; 76 ± 14%; *P* < 0.004; Wilcoxon test). The starting number of zygotes is written at the top of the one-cell bars of the histogram. (**B**) Blastocysts’ total cell numbers were similar across all groups (contralateral ICSI 114 ± 6; equatorial ICSI 108 ± 6; ipsilateral ICSI 104 ± 6; NF 110 ± 5; *P* > 0.229; Wilcoxon test). Numbers of blastocysts counted in (B): contralateral = 9; equatorial = 10; ipsilateral = 8; NF = 12. (**C**) After transfer to the uteri of pseudo-pregnant females, blastocysts from oocytes fertilized by ICSI developed to term at rates not significantly different from each other (contralateral = 34 ± 17%; equatorial = 34 ± 22%; ipsilateral = 26 ± 16%; *P* > 0.233, Wilcoxon test), albeit lower than the rates of NF counterparts (55 ± 31%; *P* > 0.073, Wilcoxon test). Starting numbers of transferred blastocysts and recipient mothers are written at the top of the leftmost bars of the histogram. Data presented in A–C are means and standard deviations; black dots represent the individual replicates.

In order to ascertain that the events observed *in vitro* were meaningful regarding those taking place *in vivo*, the pronuclear oocytes were followed up to blastocyst and to term. Accordingly, the pronuclear oocytes were cultured for 4 days inside an incubator. They progressed to blastocysts irrespective of the ICSI site, with a significant reduction in the blastocyst rate (Fig. 2A), but not in the total cell number (Fig. 2B), relative to the control embryos formed by oocytes fertilized *in vivo* and cultured *in vitro* (naturally fertilized, NF). Following the surgical transfer of the blastocysts into foster uteri of pseudo-pregnant females, birth rates were reduced, albeit not significantly, in the ICSI groups relative to the control (non-manipulated) group, particularly in the ipsilateral ICSI group (Fig. 2C). This generalized reduction of viability (particularly the drop in the developmental rate after the 8-cell stage) was probably the trade-off for the higher consistency of using the same batch of cryopreserved spermatozoa throughout the study, as it is well-known that cryopreservation is accompanied by cryodamage.

The results of this section can be summarized as follows. Site-specific ICSI is feasible, and all three fertilization sites (including the sensitive A pole of the ipsilateral ICSI) were conducive to birth. Therefore, our approach is fit for the purpose of our study: to generate two-cell embryos in which to reciprocally compare the blastomeres, knowing that their properties can be traced to only three specifically predefined sites of fertilization. In the following sections, we will refer to the pronuclear stage oocyte as a ‘zygote’, and to its first mitotic division as a ‘cleavage’. We will use the expressions ‘ICSI at the A pole’ interchangeably with ‘ipsilateral ICSI’ and ‘ICSI at the V pole’ interchangeably with ‘contralateral ICSI’ and refer to the sister blastomeres from the same embryo as ‘monozygotic.’

### Different fertilization topologies lead to distinct gene expression profiles in two-cell embryos

Given that the two models of the first zygotic cleavage (see the Introduction) were now testable with our ICSI method, we moved on to assess their effects by means of gene expression analysis. In order to focus on the effects of balanced versus unbalanced partitioning of A and V materials at the first cleavage (up to the extreme case of unbalanced partitioning, namely segregation, when cleavage is equatorial), we had to first discount a trivial possibility, namely: that the ICSI at the A or V pole, passing in the vicinity of the MII meiotic spindle (Fig. 1A and C), could *per se* induce differences of gene expression via the DNA damage response. This response can ensue due to physical damage inflicted on the spindle and, thus, on its DNA. On the contrary, this response cannot ensue in the equatorial fertilization, because the spindle does not lie on the trajectory of the ICSI needle (Fig. 1B). Therefore, we compared the three ICSI groups pairwise using the equatorial ICSI as the main reference.

**Figure 3.**
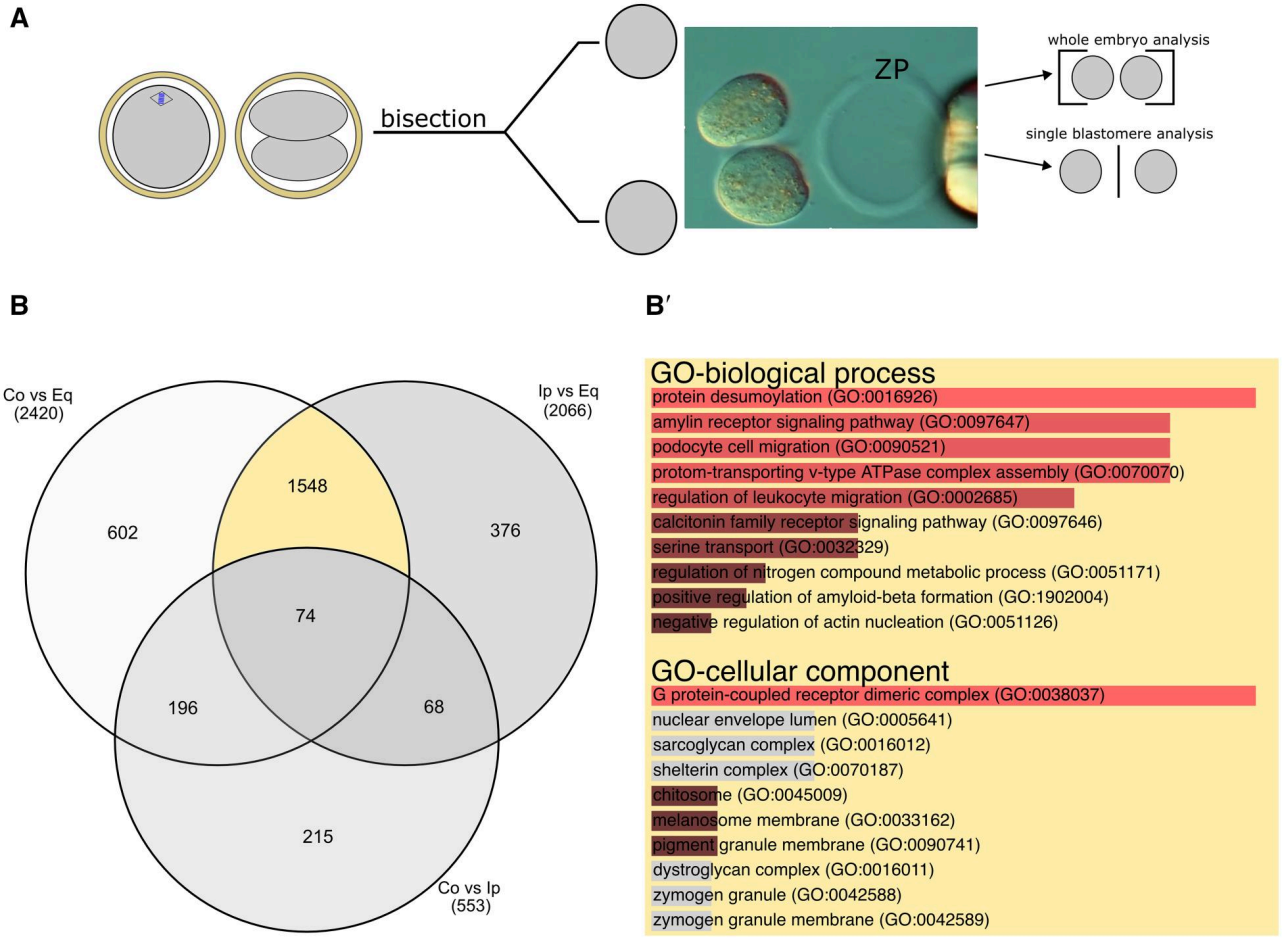
**Different fertilization topologies lead to distinct gene expression profiles in two-cell embryos**. (**A**) Scheme and representative image of a two-cell embryo being bisected at 22–24 h post-ICSI to generate single blastomeres for single cell RNA-seq (110 cells, 7492 mRNAs; GSE241089). The transcriptomes were either added together for analysis at the whole embryo level or kept distinct for analysis at the inter-blastomere level (Figs. 4 and 5). (**B**) Venn diagram representation (rendered with *InteractiVenn*; Heberle *et al.*, 2015) of the differences in gene expression between whole two-cell embryos of the three ICSI groups. (**B′**) GO terms enriched in the subset of 1548 genes representing those potentially affected by ICSI-inflicted DNA damage on the spindle (GO analysis performed with *Enrichr*; Chen *et al.*, 2013). Co, contralateral ICSI; Eq, equatorial ICSI; GO, gene ontology; Ip, ipsilateral ICSI; ZP, zona pellucida.

The early two-cell embryos were dissociated on the day following the site-specific ICSI, within 2–3 h of the first zygotic cleavage. This timing was necessary to be confident that differences between blastomeres, if any, were native and not acquired during the time elapsed from the first cleavage. Fifty pairs of blastomeres from each ICSI group (150 pairs in total) were extracted from the zona pellucida (ZP, Fig. 3A) following our established method (Casser *et al.*, 2017), and subjected to single-cell transcriptome analysis by RNA sequencing (RNA-seq). Additional pairs (production settings unchanged) were allowed to form twin blastocysts to confirm that the developmental ability was also retained beyond the two-cell stage (Supplementary Fig. S1 and Table S2). We took the following precautions aiming to minimize batch effects and day-to-day variations: (i) all three sites were injected on each ICSI day; (ii) the two-cell embryos of all three ICSI sites were bisected on the same day; (iii) the individual blastomeres were transferred to lysis buffer in a 384-well plate (Mosquito^®^) only if the PB2 had been retained inside the evacuated ZP; and (iv) the RNA extraction was conducted in parallel for all samples. In addition, the RNA-seq operator was kept blind to the fertilization site and blastomeres’ native pair associations. Following the RNA-seq, the average sequencing depth was 3.5 ± 1.3 million uniquely mapped reads per specimen of single blastomeres. As a result of quality control pipelines and filtering criteria (Materials and Methods; Supplementary Table S1), 7492 high-confidence mRNAs expressed in TPMs were retained across 110 blastomeres of 55 pairs (21, 13, and 21 pairs from the contralateral, equatorial, and ipsilateral ICSI, respectively) (GSE241089). Gene lists and TPMs are provided in Supplementary Table S3. Upon preliminary examination, we noted that transcripts known to be expressed in late but not early two-cell mouse embryos, such as *Xist* and *Zscan4*, were absent in all 110 blastomeres, which on the contrary were positive for transcripts that are expressed in early but not late mouse embryos (Deng *et al.*, 2014). This observation corroborates that the blastomeres of this study were indeed in the early phase of the two-cell stage.

In order to address whether our ICSI approach could *per se* induce differences of gene expression due to DNA damage inflicted by the ICSI on the MII spindle, we examined the differences in gene expression between the two groups in which the spindle could possibly be damaged (the contralateral and ipsilateral ICSI) and the group in which there was no such possibility (the equatorial ICSI). Since there is no reason to expect that ICSI damage (if any) would spare one of the two blastomeres, we performed this data analysis at the embryo level rather than the blastomere level; this approach also served as a way to increase the complexity of the analysis gradually as we went along, rather than immediately facing the entire complexity of the single blastomere analysis (next section). To this end, the transcript levels (TPMs) of the monozygotic blastomeres (n = 110) were added to each other for each gene (respecting the original pair associations), effectively resulting in the transcriptomes of reconstituted two-cell embryos (n = 55). These transcriptomes were compared pairwise between ICSI groups. The number of genes differently expressed (*P* < 0.05, Wilcoxon test) was greater when comparing the ICSI at either pole (ipsilateral, contralateral) with the equatorial ICSI (Fig. 3B). We defined the genes potentially affected by spindle perturbation as those that are commonly altered after the A and V pole ICSI relative to the equatorial ICSI (n = 1548 genes; marked in yellow in the Venn diagram in Fig. 3B). We next subjected this subset to GO analysis in the ontologies ‘biological process’ (BP) and ‘cellular component’ (CC). Although enrichment was found in some GO-BP and GO-CC terms, none of these was related to DNA damage and repair (Fig. 3B′). Indeed, among the most representative members of this gene family, the lowest *P* values (uncorrected for multiple comparisons) were 0.07 and 0.05 for *Brca2* and *Rad51*, respectively, in the comparison between ipsilateral and equatorial ICSI (other members of this family were unaffected regardless of which comparison; Supplementary Table S4). This overall lack of significant perturbation of genes associated with DNA damage is in line with the observation (Fig. 2B) that the blastocysts had similar cell numbers across the three groups (had DNA damage occurred, then it would have impacted the embryonic cell cycle causing the blastocyst cell numbers to be lower after the ICSI at the A or V pole).

We then asked whether there were differences of any other nature between the three ICSI groups. Examining the subset of genes that were differently expressed exclusively in the ICSI at the V pole compared to the equatorial ICSI (n = 602 genes; Fig. 3B), GO analysis returned terms broadly related to the endomembrane system (including vesicles and endoplasmic reticulum; Supplementary Fig. S2). Similar hits were returned by GO analysis of the genes which were differently expressed exclusively in the ICSI at the A pole compared to the equatorial ICSI (n = 376 genes; Fig. 3B; Supplementary Fig. S2). On the contrary, comparing the A and V pole ICSI (ipsilateral, contralateral) with each other, the differently expressed genes were fewer, and their GO analysis featured terms either spurious or inconspicuous (n = 215 genes; Fig. 3B; Supplementary Fig. S2). The endomembrane signature is reminiscent of a previous study of ours (Casser *et al.*, 2018) in which we proposed that current models were insufficient to explain the differences between the sister blastomeres of two-cell embryos after natural fertilization, and that it was necessary to invoke additional biological mechanisms possibly related to the endomembrane system. We thus asked how many of the differently expressed genes belonged in the relevant GO-CC, namely GO:0005783 (endoplasmic reticulum) and GO:0005794 (Golgi apparatus). Among the 602 genes that were differently expressed exclusively in the ICSI at the V pole compared to the equatorial ICSI, 66 and 67 were related to the endoplasmic reticulum and Golgi apparatus, respectively. Among the 376 genes that were differently expressed exclusively in the ICSI at the A pole compared to the equatorial ICSI, 54 and 48 were related to the endoplasmic reticulum and Golgi apparatus, respectively. Among the 215 genes that were differently expressed exclusively in the ICSI at the A vs. V pole, 22 and 21 were related to the endoplasmic reticulum and Golgi apparatus, respectively. As already mentioned, the two-cell embryos had developmental potential beyond that stage (Fig. 2). Therefore, for the sake of completeness, the same analysis as that of the blastomeres was applied to their derivative blastocysts. Also in this case, the analysis returned differences in gene expression related to the site of ICSI (Supplementary Fig. S1; gene lists and TPMs are provided in Supplementary Table S5). This suggests that the differences between monozygotic blastomeres can propagate to later stages.

The results of this section can be summarized as follows. The gene expression profiles of reconstituted two-cell embryos differ according to the fertilization site in a way that cannot be ascribed to DNA damage inflicted by the ICSI needle on the MII meiotic spindle. Instead—and consistent with our previous proposal (Casser *et al.*, 2018)—the fertilization sites appear to induce differences of gene expression in other families of genes, particularly in members of the endomembrane system. The cellular components of this system are unevenly localized in the ooplasm (see the Discussion) and may undergo balanced or unbalanced partitioning at mitosis according to the two competing models of the orientation of the first zygotic cleavage. We therefore examined the individual blastomeres, to see whether they would differ from each other in terms referable to the opposite regions of the ooplasm: A pole and V pole.

### Different fertilization topologies lead to distinct differences of gene expression between the monozygotic blastomeres

**Figure 4.**
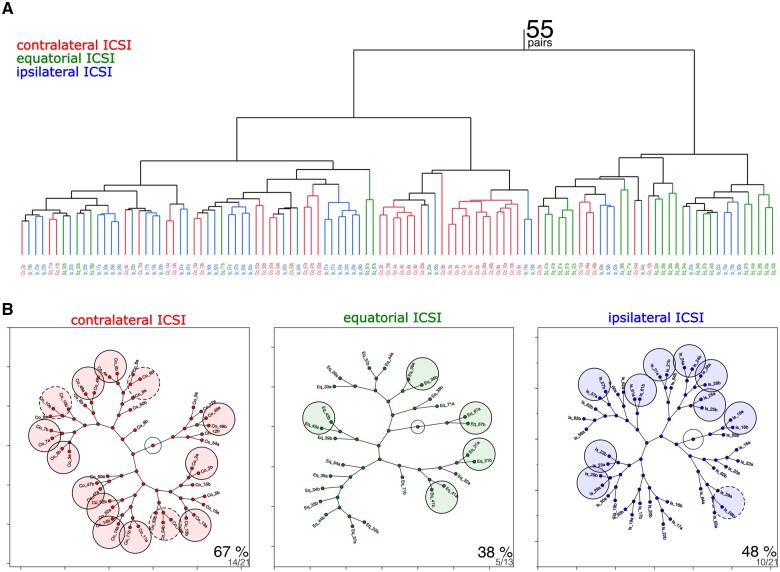
**Different fertilization topologies lead to distinct differences of gene expression between the monozygotic blastomeres**. (**A**) Following contralateral, equatorial or ipsilateral ICSI, the individual blastomeres’ transcriptomes (110 cells, 7492 mRNAs; GSE241089) were subjected to non-supervised hierarchical clustering analysis to see if they would return the original three ICSI groups. (**B**) Within the ICSI groups, the individual blastomeres’ transcriptomes were rendered as constellation diagrams to appreciate how often each blastomere would be matched correctly to the companion blastomere (encircled). Circles with solid line indicate perfect matches, circles with dotted line indicate near-perfect matches (i.e. the blastomeres and its companion blastomere are separated by a single node). The proportions shown at the bottom-right corners of constellation diagrams indicate the number of matches over the number of pairs, and the corresponding percentage. The pairs were coded as follows: ‘Co’ or ‘Eq’ or ‘Ip’ _Embryo number_‘a’ or ‘b’, to indicate the one or the other blastomere (‘a’ or ‘b’) of a given embryo (‘Embryo number’) from contralateral (Co), equatorial (Eq) or ipsilateral (Ip) ICSI.

As mentioned above, the analysis of single blastomeres was going to be more challenging, because these originate as pairs and it is not, *a priori*, possible to know which blastomere in a pair is ‘first’ and which is ‘second’. We will come back to this problem later in this section. We delved into the paired structure of the data, comparing the monozygotic blastomeres with each other as a function of the fertilization sites. We subjected the 110 individual transcriptomes to non-supervised hierarchical clustering, expecting that the blastomeres of the same embryo would be similar to each other to a greater or lesser extent depending on the ICSI site, and would, therefore, be matched with higher or lower frequency depending on the orientation of the first zygotic cleavage. At first glance the clustering returned the original three groups of the contralateral, ipsilateral, and equatorial ICSI, with few misclassifications (Fig. 4A). We then proceeded to pair the transcriptomes to see how often the *in silico* pairs would match the natural, i.e. original pair associations of the precursor two-cell embryos. We rendered the pair associations graphically by means of constellation diagrams (Fig. 4B). This graphic provides a clear and immediate view that already allowed us to note, in our previous study conducted on naturally fertilized two-cell embryos, that the monozygotic blastomeres were similar to each other in only ∼30% of the embryos (Casser *et al.*, 2017). Contrary to our previous observations, the present analysis returned 38%, 48%, and 67% (*P* = 0.08, chi square) as frequencies of correct pair associations for the ICSI at the equator, at the A pole and at the V pole, respectively. It thus appears that equatorial fertilization increases while polar (A or V) fertilization reduces the differences between monozygotic blastomeres.

**Figure 5.**
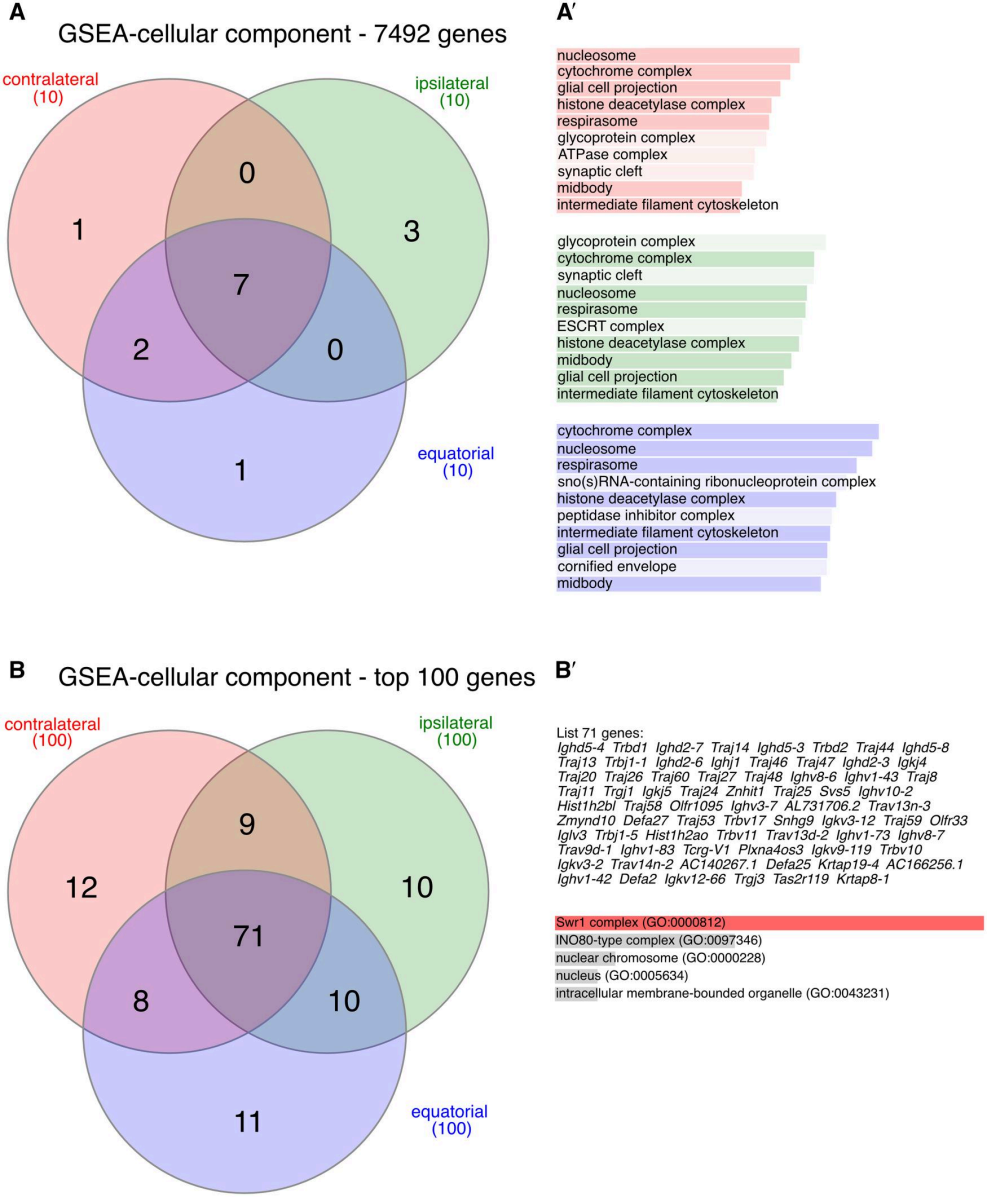
**Gene set enrichment analysis (GSEA) of the inter-blastomere differences of gene expression**. (**A**) Venn diagram representation (*InteractiVenn*; Heberle *et al.*, 2015) of the top-10 GO-CC terms found enriched (*Webgestalt*; Zhang *et al.*, 2005; Elizarraras *et al.*, 2024) in the full set (7492 mRNAs) of inter-blastomere differences (ranked) after contralateral, equatorial and ipsilateral ICSI. Seven of the 10 terms are common to the three groups. (**A′**) The seven common GO-CC terms are shown darkened in the bar chart. (**B**) Venn diagram representation of the top-100 inter-blastomere differences after contralateral, equatorial, and ipsilateral ICSI. (**B′**) The shared 71 genes were subjected to GO analysis (*Enrichr*; Chen *et al.*, 2013), returning the ‘Swr1 complex’ which is related to meiosis. GO-CC, gene ontology–cellular component.

We designed a more granular analysis to gain further insights into the possible meaning of the inter-blastomere differences as follows. We faced a problem that it is not, *a priori*, possible to know which blastomere in a pair is ‘first’ and which is ‘second’ in order to perform ratios or subtractions of transcript levels in a consistent manner across all 55 pairs of blastomeres. Even if we could know it *a posteriori* based on, for example, which blastomeres underwent the next cleavage first, the caveat is that the blastomere would no longer be at the two-cell stage. We devised a simple analytical strategy to circumvent this problem, as follows. We calculated the absolute difference of the two TPMs for each pair of blastomeres (ignoring the sign plus or minus). Using these absolute differences, it became possible to rank all genes of the dataset (7492 mRNAs) and perform gene set enrichment analysis in the GO-CC, taking into account both the rank and the absolute difference that determined the rank. Gene set enrichment analysis revealed that the inter-blastomere differences were conserved, since seven of the top ten GO-CC terms were shared among the three ICSI groups (Fig. 5A). It is of note that one of the shared GO-CC terms is the ‘midbody’ (Fig. 5A′), which is the derivative and, at the same time, the residual structure of the MII spindle after PB2 extrusion (McDougall *et al.*, 2020). To confirm, we repeated the analysis in a different way. We identified the top 100 genes with the greatest inter-blastomere differences in each of the three groups to focus on only the most differently expressed genes. Most (71%) of the inter-blastomere differences were shared (Fig. 5B). Functional enrichment analysis of the shared genes returned ‘Swr1 Complex (GO:0000812)’ as the most significant GO-CC term (Fig. 5B′), which again relates to meiosis (Gonzalez-Arranz *et al.*, 2020). As done previously, the same analysis as that of the blastomeres was applied to their derivative blastocysts. Probably due to the equalizing effect of the *in vitro* culture for 3 days, clear clusters similar to those of the blastomere analysis (Fig. 4A) could not be recognized in the blastocysts (Supplementary Fig. S1).

The results of this section can be summarized as follows. When the fertilization sites are not left to chance, but imposed by the ICSI at the A pole or V pole or equator, the differences in gene expression between monozygotic blastomeres can be consolidated into two groupings: one resulting from fertilization at either the A or V pole, after which the inter-blastomere differences are small, and the other resulted from fertilization at the equator, after which the inter-blastomere differences are greater. A common theme of these differences is that they share a meiotic signature, as if to suggest that the fertilized oocyte divides in a way that entails segregation (i.e. complete separation) of A and V hemispheres into the blastomeres of the two-cell embryo. This would be consistent with the complementarity of the molecular signatures encountered in this section (midbody, meiosis) and in the previous section (endoplasmic reticulum), since the A pole is richer in meiotic spindle-associated transcripts (VerMilyea *et al.*, 2011) while the V pole is richer in endoplasmic reticulum (Kline *et al.*, 1999; FitzHarris *et al.*, 2003). In order to shed light on this alluring possibility, we undertook the decisive experiment in the next—and last—section.

### Evidence of A–V segregation into two-cell stage blastomeres and its closer relationship to the shorter diameter of MII oocytes

**Figure 6.**
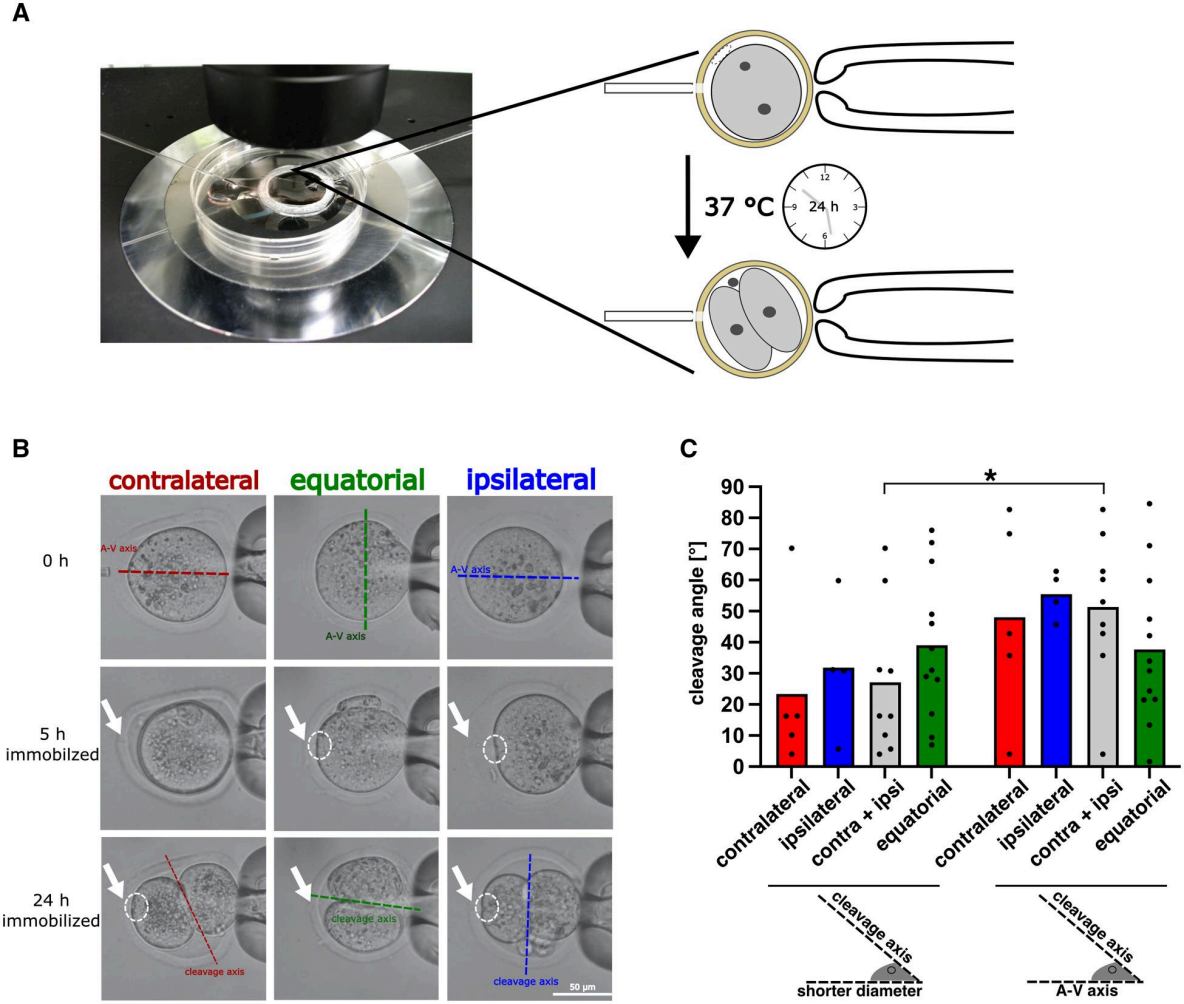
**The shorter diameter of MII oocytes overrides the effect of the fertilization site in MII mouse oocytes**. (**A**) Overview of the micromanipulation chamber placed on ThermoPlate at 37°C on the stage of the inverted microscope. One oocyte (B6C3F1) at a time was injected with a single sperm head (CD1). After injection the oocyte was not released but held in position (immobilized) for 24 h in the micromanipulation chamber, thereby allowing for pronuclear formation and first cleavage. (**B**) Representative images of oocytes of each ICSI group, showing the A–V axis of oocytes, the cleavage axis, and the ICSI hole in the zona pellucida (ZP; white arrow) as well as the dent left by the ICSI needle in the oolemma (white circle). (**C**) Distribution of angles (°) of departure of first cleavage axis from shorter diameter or from A–V axis of MII oocyte, according to the type of ICSI. The angles are smaller, i.e. the first cleavage is closer to the shorter diameter and reach statistical significance when the polar ICSIs (contra, ipsi) are combined (*P = 0.0391; Wilcoxon test).

To conclude, we wanted to see if there is any evidence for the segregation of A and V hemispheres into the blastomeres of the two-cell mouse embryo. The glitch with current approaches to the observation of the first zygotic cleavage is that the oocytes are free to move in the culture dish. They can roll together with and rotate within the ZP, to the point that some scholars have coined the expression ‘oocyte acrobatics’ (Graham *et al.*, 2021), albeit other scholars disagree on the rotation (Gardner and Davies, 2003, 2006). In order to counter these drawbacks, we secured an invariant view of MII oocytes to assess the relationship between oocyte diameters and the first cleavage axis. We held the oocytes physically immobilized, one at a time, in the micromanipulation chamber during the ICSI and for 24 h thereafter (Fig. 6A). Such follow-up was all but straightforward: in order to support development, 25% (v/v) embryo culture medium (Materials and Methods) had to be added to the micromanipulation medium and the glass floor of our standard micromanipulation chamber had to be replaced with a plastic floor (to the detriment of image quality under Nomarski optics). The physical immobilization not only secured invariant viewing, but also allowed us to relinquish the time-lapse in favour of simpler, less phototoxic static pictures. These were taken at three time points, i.e. at the time of the ICSI as well as 5 and 24 h later (Fig. 6B). The unchanged position of the ICSI hole in the ZP and the dent left by the ICSI needle in the oolemma (examples shown in Fig. 6B, white arrows and circles, respectively) attest that the oocytes did not move during the 24 h. The ICSI zygotes were followed up in development to ascertain that they were capable of reaching beyond the stage of interest (two-cell stage). The zygotes were able to progress to the 8-cell stage in the micromanipulation chamber and all the way to blastocysts when transferred to a regular incubator, where the blastocysts were also able to mimic implantation by attaching onto a layer of mitotically inactivated fibroblasts (outgrowth assay; Armant *et al.*, 1986; Kim *et al.*, 2020) (Supplementary Table S2).

With the confidence gained from the above validation, we proceeded to examine the pictures. Inspection of the pictures taken at 0 h (just prior to the ICSI) of 22 individual MII oocytes accurately oriented with pole A at 12 o’clock showed that they were not perfectly round but prolate in shape, with a longer diameter (along the A–V axis) and a shorter diameter (perpendicular to the A–V axis, i.e. equatorial). This reminded us of Hertwig’s rule, whereby cells tend to divide across the shorter diameter (Hertwig, 1884, 1893). We, therefore, examined the later pictures taken at 24 h, measuring the angle of departure of the first cleavage axis from the shorter axis and from the A–V axis (Fig. 6C). The angle was autonomous to the oocyte: when the oocyte was rotated by 90° (all other parameters unchanged), the cleavage axis also changed, following the new orientation of the oocyte, as exemplified in the middle column in Fig. 6C. The angles did not relate in any simple way to the prevalent models of the first cleavage in mice (see the Introduction). Whereas one model predicates that the first cleavage occurs parallel to the A–V axis or close to it, which is referred to as ‘meridional,’ we measured an angle of 40° to the A–V axis and 30° to the shorter axis of the MII oocyte (Fig. 6C). Whereas the other model predicts that cleavage is perpendicular to the A–V axis, i.e. equatorial only when the sperm enters the oocyte at the V pole, perpendicular cleavage also occurred after the sperm deposition at the A pole via the ipsilateral ICSI (Fig. 6C). In other words, zygotic cleavage near the equator was prevalent when the fertilization site lay on the A–V axis of the oocyte, which is a prevalence strikingly similar to that of another study in which the frequency of equatorial cleavage was quantified in 73% (Zheng *et al.*, 2013).

**Figure 7.**
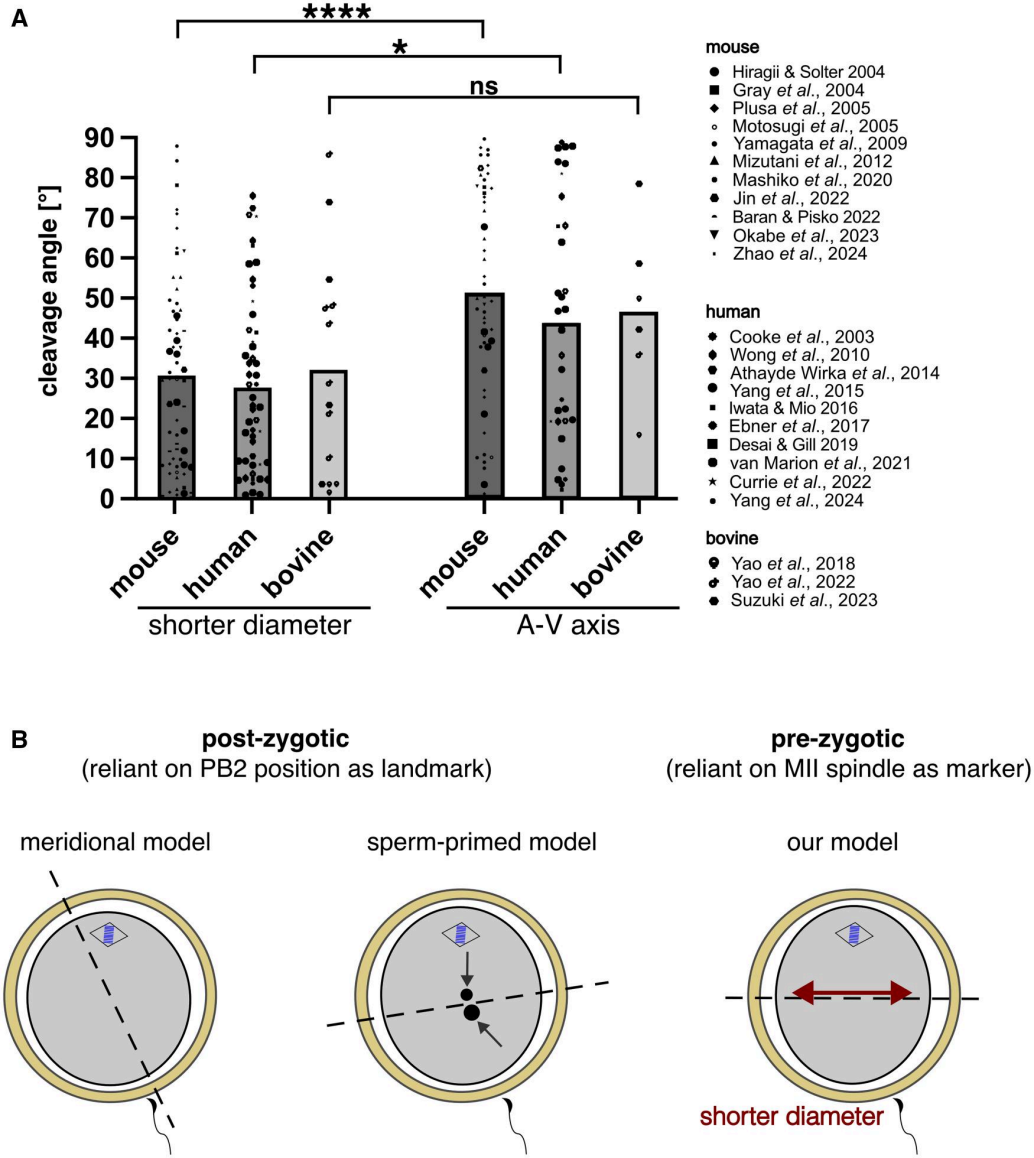
**Re-analysis of publicly available time-lapse imaging datasets and proposal for a new model of first zygotic cleavage**. (**A**) Histogram shows the angle (°) of departure of the first cleavage axis from the shorter diameter versus A–V axis of zygotes in three mammalian species. Angle (ordinate) versus species (abscissa) is shown for each scored oocyte of each study (note the symbols to the right side of the histogram). The angle of first cleavage is closer to the shorter diameter than to the A–V axis (mouse, ****P < 0.0001; human, *P = 0.0136; bovine, ns, *P* = 0.2391; Wilcoxon test). (**B**) Summary of the previous models of first zygotic cleavage (based on post-zygotic observations) compared to our proposed model (based on pre-zygotic observations). A–V, animal-vegetal; PB2, second polar body; MII, metaphase II.

We retrieved published imaging data of the first cleavage and reanalysed them to understand whether our observations had a more general validity. Our manual search of the literature returned 24 relevant studies (Fig. 7A), including 11 studies in mouse (Gray *et al.*, 2004; Hiiragi and Solter, 2004; Motosugi *et al.*, 2005; Plusa *et al.*, 2005; Yamagata *et al.*, 2009; Mizutani *et al.*, 2012; Mashiko *et al.*, 2020; Baran and Pisko, 2022; Jin *et al.*, 2022; Okabe *et al.*, 2023; Zhao *et al.*, 2024), 10 studies in human (Cooke *et al.*, 2003; Wong *et al.*, 2010; Athayde Wirka *et al.*, 2014; Yang *et al.*, 2015, 2024; Iwata and Mio, 2016; Ebner *et al.*, 2017; Desai and Gill, 2019; van Marion *et al.*, 2021; Currie *et al.*, 2022) and 3 studies in bovine (Yao *et al.*, 2018, 2022; Suzuki *et al.*, 2023). We played the movies forward until the first cleavage had occurred. At that point, we identified the shorter diameter of the zygotes and measured the angles of departure of the first cleavage axis from the shorter diameter, compared to the angles measured relative to the A–V axis. The angles of each zygote scored in each study are presented (Fig. 7A). The shorter diameter was a more reliable predictor of the orientation of the first cleavage than the A–V axis, thereby corroborating the observations enabled by our ICSI method coupled with 24-h immobilization.

The results of this section can be summarized as follows. Compared with past studies conducted on randomly positioned fertilized oocytes, our examination of MII oocytes subjected to fertilization on stage and immobilization for 24 h revealed that mouse oocytes had a strong preference to divide across their shorter diameter, apparently following Hertwig’s rule, irrespective of the fertilization site (Fig. 7B). This way of cleavage can explain why the imposition of fertilization at three different locations by the ICSI led to similar results in terms of inter-blastomere differences: the oocyte’s short diameter is one and the same regardless of the ICSI location. Moreover, it offers a rationale for the observation that discordance of twin blastocysts is preserved no matter the developmental stimulus (Casser *et al.*, 2017): again, the oocyte’s shorter diameter is one and the same.

## Discussion

This study has shown that the orientation of the first zygotic cleavage is relevant to the properties of the sister blastomeres in two-cell mouse embryos, regarding the origin of their diversity. If cleavage was meridional and fixed, then we could assume that the two-cell blastomeres are balanced in terms of their content in A and V materials and their diversification must, therefore, occur at a later stage (regulative development). If cleavage occurred at any random orientation, then we could assume that the two-cell blastomeres are unbalanced in terms of content in A and V material and, therefore, start out as different (mosaic development). In fact, we have shown here that there is also a third way: equatorial cleavage separating the A and V material into the blastomeres. This is a foundational aspect not limited to mice, but also relevant to humans since we know that the blastomeres differ from each other (Junyent *et al.*, 2024) already at the two-cell stage in not only the mouse embryo (Piotrowska *et al.*, 2001; Casser *et al.*, 2017) but also the human embryo.

The problem is that until now, it has not been clear how the zygote cleaves in the first place, with respect to its most prominent landmarks (A and V pole). We tackled this problem through a novel use of oocyte and sperm micromanipulations, building on what we think is a cytologically safer definition of the A pole, i.e. based on the MII spindle and not the PB2. Although a previous study concluded that the A pole was dispensable for full mouse development (Ciemerych *et al.*, 2000), the definition of the A pole was based on the PB2, which can be displaced from its original position and is, thus, of limited informative value. Moreover, half of the zygotes without an A pole could not develop to term (Ciemerych *et al.*, 2000), and a more recent study even claimed that mouse zygotes whose PB2 had been removed could not develop to term at all (Jin *et al.*, 2022). We did not have these concerns in our study, being based on the MII spindle as the landmark of the A pole. Our ICSI at specific and predefined ooplasmic locations (A pole, V pole, or equator) combined with blastomere dissociation at the two-cell stage is the reason why we devoted extensive controls to assure that the developmental ability was retained well beyond the developmental stage of interest. All three fertilization sites led to live births, including that at the A pole, which is usually recommended to be avoided in ICSI because of the risk of damage to the MII spindle and consequent chromosome mal-segregation (Van Der Westerlaken *et al.*, 1999; Blake *et al.*, 2000; Eichenlaub-Ritter *et al.*, 2002; Plusa *et al.*, 2002a; De los Santos *et al.*, 2016; Mori *et al.*, 2021) or, additionally, because of the risk that the sperm nucleus is dragged along with the PB2 (Mori *et al.*, 2021). Clearly, these risks are higher when the ICSI needle passes closer to the MII spindle in contralateral or ipsilateral compared to equatorial ICSI. These risks were given due consideration in the data analysis below.

We interrogated the transcriptomes of the individual sister blastomeres to clarify whether the early two-cell embryo has blastomeres that are still mirror images or have already started to diversify. Granted, a transcriptome analysis does not capture all the molecular complexity and particularly in a study such as this, with all the differences between the sister blastomeres of the two-cell embryo, however, it is a convenient starting point. Two previous transcriptomic studies could have provided useful insights into this question, were it not for the fact that they reached opposite conclusions: there were reproducible differences in the transcriptomes of sister blastomeres (Biase *et al.*, 2014) and there were no reproducible differences between sister blastomeres (VerMilyea *et al.*, 2011) of two-cell stage mouse embryos. These were generated by natural fertilization, in which the oocytes were fertilized at almost any random location on the oolemma. We did not have this confounder in our study, being based on specifically predefined sites of fertilization imposed via ICSI.

Before delving into the analysis of the transcriptomic traits traceable to the partitioning of A and V pole materials, we made sure the differences were not due to ICSI-induced DNA damage. In fairness, we observed an increased expression of DNA damage repair genes in contralateral and ipsilateral ICSI compared with equatorial ICSI; however, this increase concerned a minority of genes, and we applied no mathematical correction for multiple comparisons. Combined with the observation that the total number of embryonic cells was not reduced (Fig. 2B), as it should be if there were DNA damage (Baran and Pisko, 2022), our data support that ICSI-induced DNA damage played a marginal role in this study. It became apparent from the graphical representation of the transcriptomes by means of constellation diagrams (Fig. 4B) that the first model was not supported, as it predicted that blastomeres were mirror images, whereas the observed cases of correct matching varied in the three ICSI groups. However, the second model did not appear to be supported either, as revealed by the conserved GO signature of the inter-blastomere differences of gene expression, which contrasted with the proposed variability of the cleavage plan in this model. Compared to our initial expectation that the A pole is richer in meiotic spindle-associated transcripts (VerMilyea *et al.*, 2011) and the V pole is richer in endoplasmic reticulum (Kline *et al.*, 1999; FitzHarris *et al.*, 2003), we found that there is, indeed, a meiotic trait in the conserved GO signature. This is consistent with a study in which the PB2, which forms at the A pole after the second meiotic division, was proposed to stay attached to one of the two blastomeres and make it become different from the other blastomere by way of the material transfer from PB2 to blastomere (Jin *et al.*, 2022). The meiotic signature would make sense if the first zygotic cleavage separated the A and V poles, since the A pole contains meiosis-related transcripts surrounding the spindle (VerMilyea *et al.*, 2011), while the V pole is richer in the endoplasmic reticulum (Kline *et al.*, 1999; FitzHarris *et al.*, 2003). The problem with this proposed explanation is that if it were true, then it would imply that three distinct fertilization sites all led to the same outcome, i.e. the same separation of the A and V materials at the first cleavage.

To be able to test whether the A and V materials actually separate from each other at the first cleavage, we did something that we are not aware has ever been done before. We kept the oocytes immobilized from the time of ICSI until the first cleavage, taking pictures at the beginning and end of the immobilization. Given the novelty of this approach, we, again, devoted extensive controls to confirm that developmental ability was retained beyond the stage of interest (the blastomeres formed blastocysts, and these were able to form outgrowths in an *in vitro* implantation model). Thanks to the immobilization, we observed that the oocytes fertilized by the ICSI at the A pole (ipsilateral) or V pole (contralateral) had a strong tendency to divide across the shorter diameter, i.e. equatorially. Unlike these oocytes, those fertilized at the equator appeared to have more variability in the angle of departure of the first cleavage axis from the A–V axis. There seems to be something special about equatorial fertilization, as it was previously noted that mouse zygotes were more sensitive to the removal of equatorial material than polar material (Zernicka-Goetz, 1998). While an equatorial cleavage is detrimental for development in sea urchins (Gardner, 1996), the effects of equatorial cleavage are less clear in mammals, although it is evident that it happens (Cooke *et al.*, 2003; Gardner and Davies, 2003; Zheng *et al.*, 2013). Thanks to the immobilization of oocytes, we also noted that the equator was also the shorter diameter, albeit with exceptions. This reminded us of Hertwig’s rule (Hertwig, 1884, 1893), whereby cells tend to divide perpendicularly to their longer axis. Although response of the first cleavage axis to the shorter diameter was recognized previously in mouse zygotes either in their native state (Gardner and Davies, 2003, 2006) or when subjected to experimental lateral compression (Gray *et al.*, 2004), it is not known whether Hertwig’s rule would already apply from pre-fertilization. Notably, this seemed to have been the case—but has gone unnoticed—in previous studies of the first zygotic cleavage, as revealed in our reanalysis of 24 such studies. Thus, Hertwig’s rule could, in itself, already explain why there was a bias toward equatorial cleavage after the ICSI conducted at the A or V pole.

While advancing the conceptualization of mouse development, our study has some limitations. Our observations are contingent on the level of molecular investigation (mRNA) chosen and on the use of the ICSI, and so are the limitations. A first limitation is that transcriptomic differences between sister blastomeres do not capture the entire molecular complexity. Future work will need to look, for instance, at the global protein differences between sister blastomeres, but single-cell proteomics is presently a challenge. A second limitation is that our experiments were conducted in a single mouse strain as the oocyte donor, which is comparable to the ethnic groups in humans. Since there are differences in proteome composition between oocytes of different mouse strains (Pfeiffer *et al.*, 2015), we cannot exclude that differences of protein distribution could exist at the subcellular level as well, and these could also vary with the age of the oocyte. A third limitation is that the ICSI is not like natural fertilization or *in vitro* insemination but bypasses the normal local introduction of sperm membrane into the oocyte membrane, which some might argue could play a role in orienting cytokinesis. Indeed, the mouse oocytes fertilized via the ICSI are different from the conventionally fertilized oocytes regarding, for example, the remodelling of the egg cortical cytoskeleton and the block of polyspermy (Maleszewski *et al.*, 1996; Wortzman-Show *et al.*, 2007). A fourth and final limitation is that we did not examine the oocytes and zygotes for internal movements due to cytoplasmic streaming forces (Ajduk *et al.*, 2011; Graham *et al.*, 2021). As a consequence, the movement of the paternal pronucleus from the periphery to the centre of the ooplasm may or may not occur in a straight line (Mori *et al.*, 2021; Scheffler *et al.*, 2021), and a displacement of the paternal pronucleus could contribute to the statistical dispersion of the angles measured between the oocyte diameter (whether long or short) and the cleavage axis. Although there are limitations, the second and third do not seem to compromise the generalizability of our results, since these are in line with those obtained after natural fertilization and in different species, as shown in Fig. 7A.

In conclusion, our results attest to the contribution of MII oocyte architecture to the properties of the first two blastomeres, which originate as already different from each other rather than starting to diversify later on, in mice. Without prejudice to the notion that mammalian embryogenesis is predominantly regulative, the partitioning of oocyte territories into the blastomeres can exert an influence on the unfolding of regulative processes. This offers a rationale to explain the discordant properties of two-cell stage blastomeres and resultant monozygotic twins in mice (Casser *et al.*, 2017). Similar inter-blastomere discordance has also been observed in humans (Junyent *et al.*, 2024), thus, it is conceivable that the processes involved are conserved in the two species. In humans, ICSI and IVF are an integral part of medically assisted reproduction. It follows that no matter in which region of the ooplasm the spermatozoon may be introduced, whether by ICSI or IVF, the zygote may cleave equatorially more often than previously thought. If so, then part of the embryo developmental potential may be determined before fertilization (Yanez *et al.*, 2016) by the shape and contents of the MII oocyte. Considering that blastomeres are sometimes also removed to make a pre-implantation genetic diagnosis, removal of an early blastomere could have a greater or lesser impact depending on whether that blastomere’s cytoplasm stemmed from the A or V hemisphere of the oocyte. Some of these ideas might sound like unproven possibilities, but they are not far-fetched; they present tantalizing possibilities that could push the conceptualization of modern embryology, both basic and clinical.

## Supplementary Material

gaae045_Supplementary_Data

## Data Availability

All data needed to evaluate this article are provided in the main body or in the Supplementary Materials. The large-scale data (RNA-seq) have been deposited in the Gene Expression Omnibus of NCBI with accession number GSE241089. For convenience, the extensive information embedded in GSE241089 has also been simplified and summarized in Supplementary Tables S1, S3, and S5 (RNA-seq filtering criteria and TPM values of blastomeres and derivative blastocysts).

## References

[gaae045-B1] Ajduk A , Ilozue T, Windsor S, Yu Y, Seres KB, Bomphrey RJ, Tom BD, Swann K, Thomas A, Graham C et al Rhythmic actomyosin-driven contractions induced by sperm entry predict mammalian embryo viability. Nat Commun 2011;2:417. 21829179 10.1038/ncomms1424 PMC3265380

[gaae045-B2] Antczak M , Van Blerkom J. Oocyte influences on early development: the regulatory proteins leptin and STAT3 are polarized in mouse and human oocytes and differentially distributed within the cells of the preimplantation stage embryo. Mol Hum Reprod 1997;3:1067–1086. 9464852 10.1093/molehr/3.12.1067

[gaae045-B3] Antczak M , Van Blerkom J. Temporal and spatial aspects of fragmentation in early human embryos: possible effects on developmental competence and association with the differential elimination of regulatory proteins from polarized domains. Hum Reprod 1999;14:429–447. 10099991 10.1093/humrep/14.2.429

[gaae045-B4] Armant DR , Kaplan HA, Lennarz WJ. Fibronectin and laminin promote in vitro attachment and outgrowth of mouse blastocysts. Dev Biol 1986;116:519–523. 3732618 10.1016/0012-1606(86)90152-1

[gaae045-B5] Athayde Wirka K , Chen AA, Conaghan J, Ivani K, Gvakharia M, Behr B, Suraj V, Tan L, Shen S. Atypical embryo phenotypes identified by time-lapse microscopy: high prevalence and association with embryo development. Fertil Steril 2014;101:1637–1648 e1631-1635. 24726214 10.1016/j.fertnstert.2014.02.050

[gaae045-B6] Baran V , Pisko J. Cleavage of Early Mouse Embryo with Damaged DNA. Int J Mol Sci 2022;23:3516. 35408877 10.3390/ijms23073516 PMC8998204

[gaae045-B7] Biase FH , Cao X, Zhong S. Cell fate inclination within 2-cell and 4-cell mouse embryos revealed by single-cell RNA sequencing. Genome Res 2014;24:1787–1796. 25096407 10.1101/gr.177725.114 PMC4216920

[gaae045-B8] Blake M , Garrisi J, Tomkin G, Cohen J. Sperm deposition site during ICSI affects fertilization and development. Fertil Steril 2000;73:31–37. 10632408 10.1016/s0015-0282(99)00465-3

[gaae045-B9] Boiani M , Casser E, Fuellen G, Christians ES. Totipotency continuity from zygote to early blastomeres: a model under revision. Reproduction 2019;158:R49–R65. 30978695 10.1530/REP-18-0462

[gaae045-B10] Boiani M , Eckardt S, Scholer HR, McLaughlin KJ. Oct4 distribution and level in mouse clones: consequences for pluripotency. Genes Dev 2002;16:1209–1219. 12023300 10.1101/gad.966002 PMC186284

[gaae045-B11] Casser E , Israel S, Schlatt S, Nordhoff V, Boiani M. Retrospective analysis: reproducibility of interblastomere differences of mRNA expression in 2-cell stage mouse embryos is remarkably poor due to combinatorial mechanisms of blastomere diversification. Mol Hum Reprod 2018;24:388–400. 29746690 10.1093/molehr/gay021

[gaae045-B12] Casser E , Israel S, Witten A, Schulte K, Schlatt S, Nordhoff V, Boiani M. Totipotency segregates between the sister blastomeres of two-cell stage mouse embryos. Sci Rep 2017;7:8299. 28811525 10.1038/s41598-017-08266-6 PMC5557898

[gaae045-B13] Cavaleri F , Gentile L, Scholer HR, Boiani M. Recombinant human albumin supports development of somatic cell nuclear transfer embryos in mice: toward the establishment of a chemically defined cloning protocol. Cloning Stem Cells 2006;8:24–40. 16571075 10.1089/clo.2006.8.24

[gaae045-B14] Chen EY , Tan CM, Kou Y, Duan Q, Wang Z, Meirelles GV, Clark NR, Ma’ayan A. Enrichr: interactive and collaborative HTML5 gene list enrichment analysis tool. BMC Bioinformatics 2013;14:128. 23586463 10.1186/1471-2105-14-128 PMC3637064

[gaae045-B15] Ciemerych MA , Mesnard D, Zernicka-Goetz M. Animal and vegetal poles of the mouse egg predict the polarity of the embryonic axis, yet are nonessential for development. Development 2000;127:3467–3474. 10903172 10.1242/dev.127.16.3467

[gaae045-B16] Cooke S , Tyler JP, Driscoll GL. Meiotic spindle location and identification and its effect on embryonic cleavage plane and early development. Hum Reprod 2003;18:2397–2405. 14585893 10.1093/humrep/deg447

[gaae045-B17] Currie CE , Ford E, Benham Whyte L, Taylor DM, Mihalas BP, Erent M, Marston AL, Hartshorne GM, McAinsh AD. The first mitotic division of human embryos is highly error prone. Nat Commun 2022;13:6755. 36347869 10.1038/s41467-022-34294-6 PMC9643329

[gaae045-B18] Dalcq AM. Introduction to General Embryology. London, UK: Oxford University Press, 1957.

[gaae045-B19] De los Santos MJ , Apter S, Coticchio G, Debrock S, Lundin K, Plancha CE, Prados F, Rienzi L, Verheyen G, Woodward B et al; ESHRE Guideline Group on Good Practice in IVF Labs. Revised guidelines for good practice in IVF laboratories (2015). Hum Reprod 2016;31:685–686. 26908842 10.1093/humrep/dew016

[gaae045-B20] Deng Q , Ramskold D, Reinius B, Sandberg R. Single-cell RNA-seq reveals dynamic, random monoallelic gene expression in mammalian cells. Science 2014;343:193–196. 24408435 10.1126/science.1245316

[gaae045-B21] Denker HW. Cell lineage, determination and differentiation in earliest developmental stages in mammals. Bibl Anat 1983;24:22–58. 6342603

[gaae045-B22] Desai N , Gill P. Blastomere cleavage plane orientation and the tetrahedral formation are associated with increased probability of a good-quality blastocyst for cryopreservation or transfer: a time-lapse study. Fertil Steril 2019;111:1159–1168.e1. 30982605 10.1016/j.fertnstert.2019.02.019

[gaae045-B23] Driesch H. The potency of the first two cleavage cells in Echinoderm development. Experimental production of partial and double formations. In: Willier BH, Oppenheimer JM (eds). Foundations of Experimental Embryology. Englewood Cliffs, NJ: Prentice-Hall, 1964 (1892), 38–51.

[gaae045-B24] Duncan FE , Moss SB, Schultz RM, Williams CJ. PAR-3 defines a central subdomain of the cortical actin cap in mouse eggs. Dev Biol 2005;280:38–47. 15766746 10.1016/j.ydbio.2004.12.034

[gaae045-B25] Ebner T , Hoggerl A, Oppelt P, Radler E, Enzelsberger SH, Mayer RB, Petek E, Shebl O. Time-lapse imaging provides further evidence that planar arrangement of blastomeres is highly abnormal. Arch Gynecol Obstet 2017;296:1199–1205. 28932956 10.1007/s00404-017-4531-5

[gaae045-B26] Edwards RG. Genetics of polarity in mammalian embryos. Reprod Biomed Online 2005;11:104–114. 16102297 10.1016/s1472-6483(10)61305-3

[gaae045-B27] Eichenlaub-Ritter U , Shen Y, Tinneberg HR. Manipulation of the oocyte: possible damage to the spindle apparatus. Reprod Biomed Online 2002;5:117–124. 12419035 10.1016/s1472-6483(10)61613-6

[gaae045-B28] Elizarraras JM , Liao Y, Shi Z, Zhu Q, Pico AR, Zhang B. WebGestalt 2024: faster gene set analysis and new support for metabolomics and multi-omics. Nucleic Acids Res 2024;52:W415–W421. 38808672 10.1093/nar/gkae456 PMC11223849

[gaae045-B29] Evans JP , Foster JA, McAvey BA, Gerton GL, Kopf GS, Schultz RM. Effects of perturbation of cell polarity on molecular markers of sperm-egg binding sites on mouse eggs. Biol Reprod 2000;62:76–84. 10611070 10.1095/biolreprod62.1.76

[gaae045-B30] FitzHarris G , Marangos P, Carroll J. Cell cycle-dependent regulation of structure of endoplasmic reticulum and inositol 1,4,5-trisphosphate-induced Ca2+ release in mouse oocytes and embryos. Mol Biol Cell 2003;14:288–301. 12529444 10.1091/mbc.E02-07-0431 PMC140245

[gaae045-B31] Fraser LR , Drury LM. The relationship between sperm concentration and fertilization in vitro of mouse eggs. Biol Reprod 1975;13:513–518. 1203407 10.1095/biolreprod13.5.513

[gaae045-B32] Gardner RL. Can developmentally significant spatial patterning of the egg be discounted in mammals? Hum Reprod Update 1996;2:3–27. 9079400 10.1093/humupd/2.1.3

[gaae045-B33] Gardner RL. Scrambled or bisected mouse eggs and the basis of patterning in mammals. Bioessays 1999;21:271–274. 10377889 10.1002/(SICI)1521-1878(199904)21:4<271::AID-BIES2>3.0.CO;2-C

[gaae045-B34] Gardner RL , Davies TJ. The basis and significance of pre-patterning in mammals. Philos Trans R Soc Lond B Biol Sci 2003;358:1331–1338. discussion 1338–1339. 14511479 10.1098/rstb.2003.1322 PMC1693237

[gaae045-B35] Gardner RL , Davies TJ. An investigation of the origin and significance of bilateral symmetry of the pronuclear zygote in the mouse. Hum Reprod 2006;21:492–502. 16210387 10.1093/humrep/dei318

[gaae045-B36] Gonzalez-Arranz S , Gardner JM, Yu Z, Patel NJ, Heldrich J, Santos B, Carballo JA, Jaspersen SL, Hochwagen A, San-Segundo PA. SWR1-independent association of H2A.Z to the LINC complex promotes meiotic chromosome motion. Front Cell Dev Biol 2020;8:594092. 33195270 10.3389/fcell.2020.594092 PMC7642583

[gaae045-B37] Graham CF , Windsor S, Ajduk A, Trinh T, Vincent A, Jones C, Coward K, Kalsi D, Zernicka-Goetz M, Swann K et al Dynamic shapes of the zygote and two-cell mouse and human. Biol Open 2021;10:bio059013. 10.1242/bio.059013 PMC8713988 34935907

[gaae045-B38] Gray D , Plusa B, Piotrowska K, Na J, Tom B, Glover DM, Zernicka-Goetz M. First cleavage of the mouse embryo responds to change in egg shape at fertilization. Curr Biol 2004;14:397–405. 15028215 10.1016/j.cub.2004.02.031

[gaae045-B39] Heberle H , Meirelles GV, da Silva FR, Telles GP, Minghim R. InteractiVenn: a web-based tool for the analysis of sets through Venn diagrams. BMC Bioinformatics 2015;16:169. 25994840 10.1186/s12859-015-0611-3 PMC4455604

[gaae045-B40] Hertwig O. Das Problem der Befruchtung und der Isotropie des Eies. Eine Theorie der Vererbung. Jenaische Zeitschrift für Naturwissenschaft 1884;18:276–318.

[gaae045-B41] Hertwig O. Ueber den Werth der ersten Furchungszellen für die Organbildung des Embryo Experimentelle Studien am Frosch-und Tritonei. Archiv f Mikrosk Anat 1893;42:662–807.

[gaae045-B42] Hiiragi T , Solter D. First cleavage plane of the mouse egg is not predetermined but defined by the topology of the two apposing pronuclei. Nature 2004;430:360–364. 15254539 10.1038/nature02595

[gaae045-B43] Hupalowska A , Jedrusik A, Zhu M, Bedford MT, Glover DM, Zernicka-Goetz M. CARM1 and paraspeckles regulate pre-implantation mouse embryo development. Cell 2018;175:1902–1916.e13. e1913. 30550788 10.1016/j.cell.2018.11.027 PMC6292842

[gaae045-B44] Iwata K , Mio Y. Observation of human embryonic behavior in vitro by high-resolution time-lapse cinematography. Reprod Med Biol 2016;15:145–154. 29259431 10.1007/s12522-015-0231-7 PMC5715854

[gaae045-B45] Jin H , Han Y, Wang H, Li JXH, Shen W, Zhang L, Chen L, Jia S, Yuan P, Chen H et al The second polar body contributes to the fate asymmetry in the mouse embryo. Natl Sci Rev 2022;9:nwac003. 35919785 10.1093/nsr/nwac003 PMC9337984

[gaae045-B46] Junyent S , Meglicki M, Vetter R, Mandelbaum R, King C, Patel EM, Iwamoto-Stohl L, Reynell C, Chen DY, Rubino P et al The first two blastomeres contribute unequally to the human embryo. Cell 2024;187:2838–2854.e17. 38744282 10.1016/j.cell.2024.04.029

[gaae045-B47] Kim J , Lee J, Jun JH. Advantages of the outgrowth model for evaluating the implantation competence of blastocysts. Clin Exp Reprod Med 2020;47:85–93. 32521581 10.5653/cerm.2019.03216 PMC7315857

[gaae045-B48] Kimura Y , Yanagimachi R. Intracytoplasmic sperm injection in the mouse. Biol Reprod 1995;52:709–720. 7779992 10.1095/biolreprod52.4.709

[gaae045-B49] Kline D , Mehlmann L, Fox C, Terasaki M. The cortical endoplasmic reticulum (ER) of the mouse egg: localization of ER clusters in relation to the generation of repetitive calcium waves. Dev Biol 1999;215:431–442. 10545249 10.1006/dbio.1999.9445

[gaae045-B50] Krawczyk K , Kosyl E, Częścik-Łysyszyn K, Wyszomirski T, Maleszewski M. Developmental capacity is unevenly distributed among single blastomeres of 2-cell and 4-cell stage mouse embryos. Sci Rep 2021;11:21422. 34728646 10.1038/s41598-021-00834-1 PMC8563712

[gaae045-B51] Lawrence M , Huber W, Pages H, Aboyoun P, Carlson M, Gentleman R, Morgan MT, Carey VJ. Software for computing and annotating genomic ranges. PLoS Comput Biol 2013;9:e1003118. 23950696 10.1371/journal.pcbi.1003118 PMC3738458

[gaae045-B52] Littwin T , Denker HW. Segregation during cleavage in the mammalian embryo? A critical comparison of whole-mount/CLSM and section immunohistochemistry casts doubts on segregation of axis-relevant leptin domains in the rabbit. Histochem Cell Biol 2011;135:553–570. 21626127 10.1007/s00418-011-0816-0

[gaae045-B53] Maemura M , Taketsuru H, Nakajima Y, Shao R, Kakihara A, Nogami J, Ohkawa Y, Tsukada YI. Totipotency of mouse zygotes extends to single blastomeres of embryos at the four-cell stage. Sci Rep 2021;11:11167. 34045607 10.1038/s41598-021-90653-1 PMC8160171

[gaae045-B54] Maleszewski M , Kimura Y, Yanagimachi R. Sperm membrane incorporation into oolemma contributes to the oolemma block to sperm penetration: evidence based on intracytoplasmic sperm injection experiments in the mouse. Mol Reprod Dev 1996;44:256–259. 9115725 10.1002/(SICI)1098-2795(199606)44:2<256::AID-MRD16>3.0.CO;2-0

[gaae045-B55] Mashiko D , Ikeda Z, Yao T, Tokoro M, Fukunaga N, Asada Y, Yamagata K. Chromosome segregation error during early cleavage in mouse pre-implantation embryo does not necessarily cause developmental failure after blastocyst stage. Sci Rep 2020;10:854. 31965014 10.1038/s41598-020-57817-x PMC6972754

[gaae045-B56] McDougall A , Hebras C, Pruliere G, Burgess D, Costache V, Dumollard R, Chenevert J. Role of PB1 midbody remnant creating tethered polar bodies during meiosis II. Genes (Basel) 2020;11:1394. 33255457 10.3390/genes11121394 PMC7760350

[gaae045-B57] Mizutani E , Yamagata K, Ono T, Akagi S, Geshi M, Wakayama T. Abnormal chromosome segregation at early cleavage is a major cause of the full-term developmental failure of mouse clones. Dev Biol 2012;364:56–65. 22266425 10.1016/j.ydbio.2012.01.001

[gaae045-B58] Mori M , Yao T, Mishina T, Endoh H, Tanaka M, Yonezawa N, Shimamoto Y, Yonemura S, Yamagata K, Kitajima TS et al RanGTP and the actin cytoskeleton keep paternal and maternal chromosomes apart during fertilization. J Cell Biol 2021;220:e202012001. 10.1083/jcb.202012001 PMC8404465 34424312

[gaae045-B59] Morris SA , Guo Y, Zernicka-Goetz M. Developmental plasticity is bound by pluripotency and the Fgf and Wnt signaling pathways. Cell Rep 2012;2:756–765. 23041313 10.1016/j.celrep.2012.08.029 PMC3607220

[gaae045-B60] Motosugi N , Bauer T, Polanski Z, Solter D, Hiiragi T. Polarity of the mouse embryo is established at blastocyst and is not prepatterned. Genes Dev 2005;19:1081–1092. 15879556 10.1101/gad.1304805 PMC1091742

[gaae045-B61] Okabe M , Shirasawa H, Ono Y, Goto M, Iwasawa T, Sakaguchi T, Fujishima A, Onodera Y, Makino K, Miura H et al An approach for live imaging of first cleavage in mouse embryos using fluorescent chemical probes for DNA, microtubules, and microfilaments. Reprod Med Biol 2023;22:e12551. 38023339 10.1002/rmb2.12551 PMC10680128

[gaae045-B62] Pfeiffer MJ , Taher L, Drexler H, Suzuki Y, Makałowski W, Schwarzer C, Wang B, Fuellen G, Boiani M. Differences in embryo quality are associated with differences in oocyte composition: a proteomic study in inbred mice. Proteomics 2015;15:675–687. 25367296 10.1002/pmic.201400334

[gaae045-B63] Piotrowska K , Wianny F, Pedersen RA, Zernicka-Goetz M. Blastomeres arising from the first cleavage division have distinguishable fates in normal mouse development. Development 2001;128:3739–3748. 11585800 10.1242/dev.128.19.3739

[gaae045-B64] Piotrowska K , Zernicka-Goetz M. Role for sperm in spatial patterning of the early mouse embryo. Nature 2001;409:517–521. 11206548 10.1038/35054069

[gaae045-B65] Piotrowska-Nitsche K , Chan AW. Effect of sperm entry on blastocyst development after in vitro fertilization and intracytoplasmic sperm injection—mouse model. J Assist Reprod Genet 2013;30:81–89. 23224695 10.1007/s10815-012-9896-6 PMC3553354

[gaae045-B66] Plusa B , Grabarek JB, Piotrowska K, Glover DM, Zernicka-Goetz M. Site of the previous meiotic division defines cleavage orientation in the mouse embryo. Nat Cell Biol 2002a;4:811–815. 12360292 10.1038/ncb860

[gaae045-B67] Plusa B , Hadjantonakis AK, Gray D, Piotrowska-Nitsche K, Jedrusik A, Papaioannou VE, Glover DM, Zernicka-Goetz M. The first cleavage of the mouse zygote predicts the blastocyst axis. Nature 2005;434:391–395. 15772664 10.1038/nature03388

[gaae045-B68] Plusa B , Piotrowska K, Zernicka-Goetz M. Sperm entry position provides a surface marker for the first cleavage plane of the mouse zygote. Genesis 2002b;32:193–198. 11892007 10.1002/gene.10027

[gaae045-B69] Scheffler K , Uraji J, Jentoft I, Cavazza T, Monnich E, Mogessie B, Schuh M. Two mechanisms drive pronuclear migration in mouse zygotes. Nat Commun 2021;12:841. 33547291 10.1038/s41467-021-21020-x PMC7864974

[gaae045-B70] Schindelin J , Arganda-Carreras I, Frise E, Kaynig V, Longair M, Pietzsch T, Preibisch S, Rueden C, Saalfeld S, Schmid B et al Fiji: an open-source platform for biological-image analysis. Nat Methods 2012;9:676–682. 22743772 10.1038/nmeth.2019 PMC3855844

[gaae045-B71] Schulz LC , Roberts RM. Dynamic changes in leptin distribution in the progression from ovum to blastocyst of the pre-implantation mouse embryo. Reproduction 2011;141:767–777. 21444625 10.1530/REP-10-0532 PMC3214761

[gaae045-B72] Seidel F. Die Entwicklungspotenzen einer isolierten Blastomere des Zweizellenstadiums im Säugetierei. Naturwissenschaften 1952;39:355–356.

[gaae045-B73] Seidel F. Die Entwicklungsfähigkeiten isolierter Furchungszellen aus dem Ei des Kaninchens Oryctolagus cuniculus. W Roux’ Archiv Entwicklungsmechanik 1960;152:43–130. 10.1007/BF00575220 28354918

[gaae045-B74] Simerly CR , Takahashi D, Jacoby E, Castro C, Hartnett C, Hewitson L, Navara C, Schatten G. Fertilization and cleavage axes differ in primates conceived by conventional (IVF) versus intracytoplasmic sperm injection (ICSI). Sci Rep 2019;9:15282. 31653971 10.1038/s41598-019-51815-4 PMC6814755

[gaae045-B5621274] Summers MC, , McGinnis LK, , Lawitts JA, , Raffin M, , Biggers JD. IVF of mouse ova in a simplex optimized medium supplemented with amino acids. Hum Reprod 2000;15:1791–1801. 10920105 10.1093/humrep/15.8.1791

[gaae045-B75] Suzuki R , Yao T, Okada M, Nagai H, Khurchabilig A, Kobayashi J, Yamagata K, Sugimura S. Direct cleavage during the first mitosis is a sign of abnormal fertilization in cattle. Theriogenology 2023;200:96–105. 36805250 10.1016/j.theriogenology.2023.01.028

[gaae045-B76] Tam PPL. First mitotic division: getting it right at the start. Nat Cell Biol 2002;4:E232. 12360303 10.1038/ncb1002-e232

[gaae045-B77] Tarkowski AK. Experiments on the development of isolated blastomers of mouse eggs. Nature 1959;184:1286–1287. 13836947 10.1038/1841286a0

[gaae045-B78] Van Der Westerlaken LA , Helmerhorst FM, Hermans J, Naaktgeboren N. Intracytoplasmic sperm injection: position of the polar body affects pregnancy rate. Hum Reprod 1999;14:2565–2569. 10527988 10.1093/humrep/14.10.2565

[gaae045-B79] van Marion ES , Speksnijder JP, Hoek J, Boellaard WPA, Dinkelman-Smit M, Chavli EA, Steegers-Theunissen RPM, Laven JSE, Baart EB. Time-lapse imaging of human embryos fertilized with testicular sperm reveals an impact on the first embryonic cell cycle. Biol Reprod 2021;104:1218–1227. 33690817 10.1093/biolre/ioab031 PMC8181962

[gaae045-B80] VerMilyea MD , Maneck M, Yoshida N, Blochberger I, Suzuki E, Suzuki T, Spang R, Klein CA, Perry AC. Transcriptome asymmetry within mouse zygotes but not between early embryonic sister blastomeres. EMBO J 2011;30:1841–1851. 21468028 10.1038/emboj.2011.92 PMC3101998

[gaae045-B81] Wang J , Wang L, Feng G, Wang Y, Li Y, Li X, Liu C, Jiao G, Huang C, Shi J et al Asymmetric expression of LincGET biases cell fate in two-cell mouse embryos. Cell 2018;175:1887–1901.e18. e1818. 30550787 10.1016/j.cell.2018.11.039

[gaae045-B82] Wang Y , Zheng X, Cheng R, Han J, Ma X, Xu W, Gao L, Lei A, Liu J, Quan F et al Asymmetric expression of maternal mRNA governs first cell-fate decision. FASEB J 2021;35:e22006. 34694646 10.1096/fj.202101196R

[gaae045-B83] Wolf FA , Angerer P, Theis FJ. SCANPY: large-scale single-cell gene expression data analysis. Genome Biol 2018;19:15. 29409532 10.1186/s13059-017-1382-0 PMC5802054

[gaae045-B84] Wong CC , Loewke KE, Bossert NL, Behr B, De Jonge CJ, Baer TM, Reijo Pera RA. Non-invasive imaging of human embryos before embryonic genome activation predicts development to the blastocyst stage. Nat Biotechnol 2010;28:1115–1121. 20890283 10.1038/nbt.1686

[gaae045-B85] Wortzman-Show GB , Kurokawa M, Fissore RA, Evans JP. Calcium and sperm components in the establishment of the membrane block to polyspermy: studies of ICSI and activation with sperm factor. Mol Hum Reprod 2007;13:557–565. 17575288 10.1093/molehr/gam042

[gaae045-B86] Yamagata K , Suetsugu R, Wakayama T. Assessment of chromosomal integrity using a novel live-cell imaging technique in mouse embryos produced by intracytoplasmic sperm injection. Hum Reprod 2009;24:2490–2499. 19574276 10.1093/humrep/dep236

[gaae045-B87] Yanez LZ , Han J, Behr BB, Pera RAR, Camarillo DB. Human oocyte developmental potential is predicted by mechanical properties within hours after fertilization. Nat Commun 2016;7:10809. 26904963 10.1038/ncomms10809 PMC4770082

[gaae045-B88] Yang HY , Leahy BD, Jang WD, Wei D, Kalma Y, Rahav R, Carmon A, Kopel R, Azem F, Venturas M et al BlastAssist: a deep learning pipeline to measure interpretable features of human embryos. Hum Reprod 2024;39:698–708. 38396213 10.1093/humrep/deae024 PMC11648949

[gaae045-B89] Yang ST , Shi JX, Gong F, Zhang SP, Lu CF, Tan K, Leng LZ, Hao M, He H, Gu YF et al Cleavage pattern predicts developmental potential of day 3 human embryos produced by IVF. Reprod Biomed Online 2015;30:625–634. 25892500 10.1016/j.rbmo.2015.02.008

[gaae045-B90] Yao T , Suzuki R, Furuta N, Suzuki Y, Kabe K, Tokoro M, Sugawara A, Yajima A, Nagasawa T, Matoba S et al Live-cell imaging of nuclear-chromosomal dynamics in bovine in vitro fertilised embryos. Sci Rep 2018;8:7460. 29748644 10.1038/s41598-018-25698-w PMC5945782

[gaae045-B91] Yao T , Ueda A, Khurchabilig A, Mashiko D, Tokoro M, Nagai H, Sho T, Matoba S, Yamagata K, Sugimura S. Micronucleus formation during early cleavage division is a potential hallmark of preimplantation embryonic loss in cattle. Biochem Biophys Res Commun 2022;617:25–32. 35689839 10.1016/j.bbrc.2022.05.075

[gaae045-B92] Zernicka-Goetz M. Fertile offspring derived from mammalian eggs lacking either animal or vegetal poles. Development 1998;125:4803–4808. 9806928 10.1242/dev.125.23.4803

[gaae045-B93] Zhang B , Kirov S, Snoddy J. WebGestalt: an integrated system for exploring gene sets in various biological contexts. Nucleic Acids Res 2005;33:W741–748. 15980575 10.1093/nar/gki475 PMC1160236

[gaae045-B94] Zhao Y , Zhang M, Liu J, Hu X, Sun Y, Huang X, Li J, Lei L. Nr5a2 ensures inner cell mass formation in mouse blastocyst. Cell Rep 2024;43:113840. 38386558 10.1016/j.celrep.2024.113840

[gaae045-B95] Zheng JG , Huo T, Chen T, Wang C, Zhang N, Tian N, Zhao F, Lu D, Chen D, Ma W et al Understanding three-dimensional spatial relationship between the mouse second polar body and first cleavage plane with full-field optical coherence tomography. J Biomed Opt 2013;18:10503. 23238420 10.1117/1.JBO.18.1.010503

