# Supplementary material for: Animal and vegetal materials of mouse oocytes segregate at first zygotic cleavage: a simple mechanism that makes the two-cell blastomeres differ reciprocally from the start
Source: Mol Hum Reprod. 2024 Dec 30;31(1):gaae045. doi: 10.1093/molehr/gaae045 (PMC11741683; doi:10.1093/molehr/gaae045)
Supplement: gaae045_Supplementary_Data [file gaae045_supplementary_data.zip › b12e6_Nolte et al_supplementary materials_R1.pdf]

Supplementary information files

Animal and vegetal materials of mouse oocytes segregate at first zygotic cleavage: a simple mechanism that makes the 2-cell blastomeres differ reciprocally from the start

Thomas Nolte, Reza Halabian, Steffen Israel, Yutaka Suzuki, Roberto A. Avelar, Daniel Palmer, Georg Fuellen, Wojciech Makalowski, Michele Boiani

Supplementary figure S1. Analysis of twin blastocysts obtained from monozygotic blastomeres.
Supplementary figure S2. Further evidence that different fertilization topologies lead to distinct gene expression profiles in 2-cell embryos.

Supplementary table S1. Overview of the number of analyzed genes in this transcriptome study of single blastomeres and blastocysts, with summary of the filtering criteria applied to the RNA-seq data.
Supplementary table S2. Developmental validation of ICSI zygote viability after 24 h immobilization.
Supplementary table S3. TPM values of the single blastomeres (55 pairs, 7492 genes).
Supplementary table S4. Transcripts of DNA-damage repair genes detected in this study.
Supplementary table S5. TPM values of intact and twin blastocysts (19 pairs, 7660 genes).

Supplementary figures

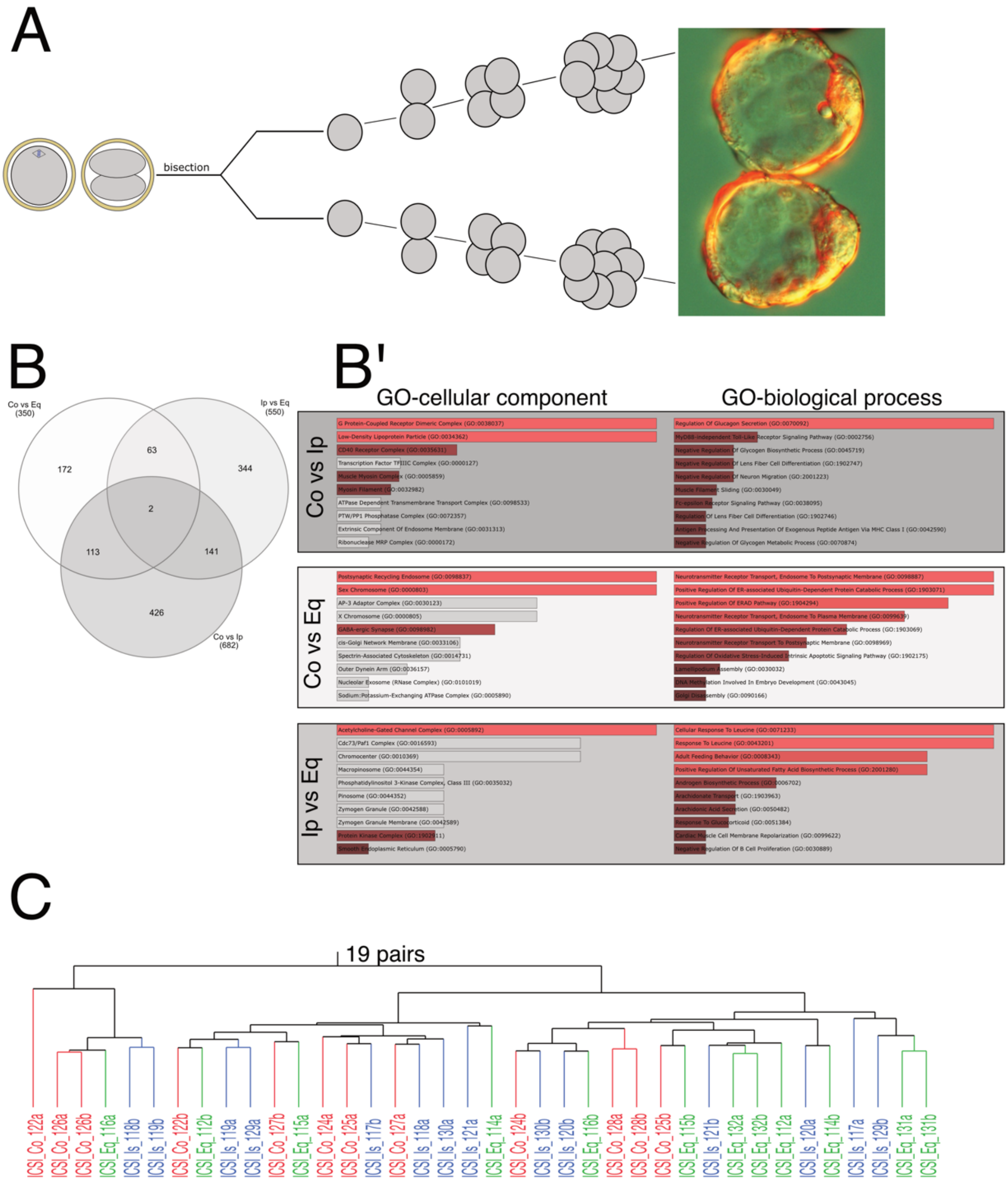

Supplementary figure S1. Analysis of twin blastocysts obtained from monozygotic blastomeres. Fourteen pairs of blastocysts from each ICSI group (total n = 42) were processed for RNA-seq, with an average sequencing depth 2.9 ± 1.1 million uniquely mapped reads per specimen of single blastocysts. After applying the same pipeline and the same quality control criteria as for the blastomeres (Supplementary Table S1), 7660 mRNAs were retained across 6, 7 and 6 pairs (total pairs n = 19) of twin blastocysts from the contralateral, ipsilateral and equatorial group, respectively (GSE241089, Supplementary Table S3). **(A).** Scheme and representative image of twin blastocysts from a split 2-cell embryo. **(B).** Venn diagram representation (rendered with *InteractiVenn*; Heberle *et al.*, 2015) of the differences of gene expression between twin blastocysts of the three ICSI groups. **(B').** The differently expressed genes specific to each comparison (contralateral vs ipsilateral ICSI, contralateral vs equatorial ICSI, ipsilateral vs equatorial ICSI) were subjected to GO analysis using *Enrichr* (Chen *et al.*, 2013). **(C).** The blastocysts' transcriptomes were subjected to non-supervised hierarchical clustering analysis to see if they would return the original three ICSI groups. The coding system is as follows: ICSI_'Co' or 'Eq' or 'Is' _Embryo number_'a' or 'b', to indicate the one or the other twin ('a' or 'b') of a given embryo ('Embryo number') from contralateral, equatorial or ipsilateral ICSI.

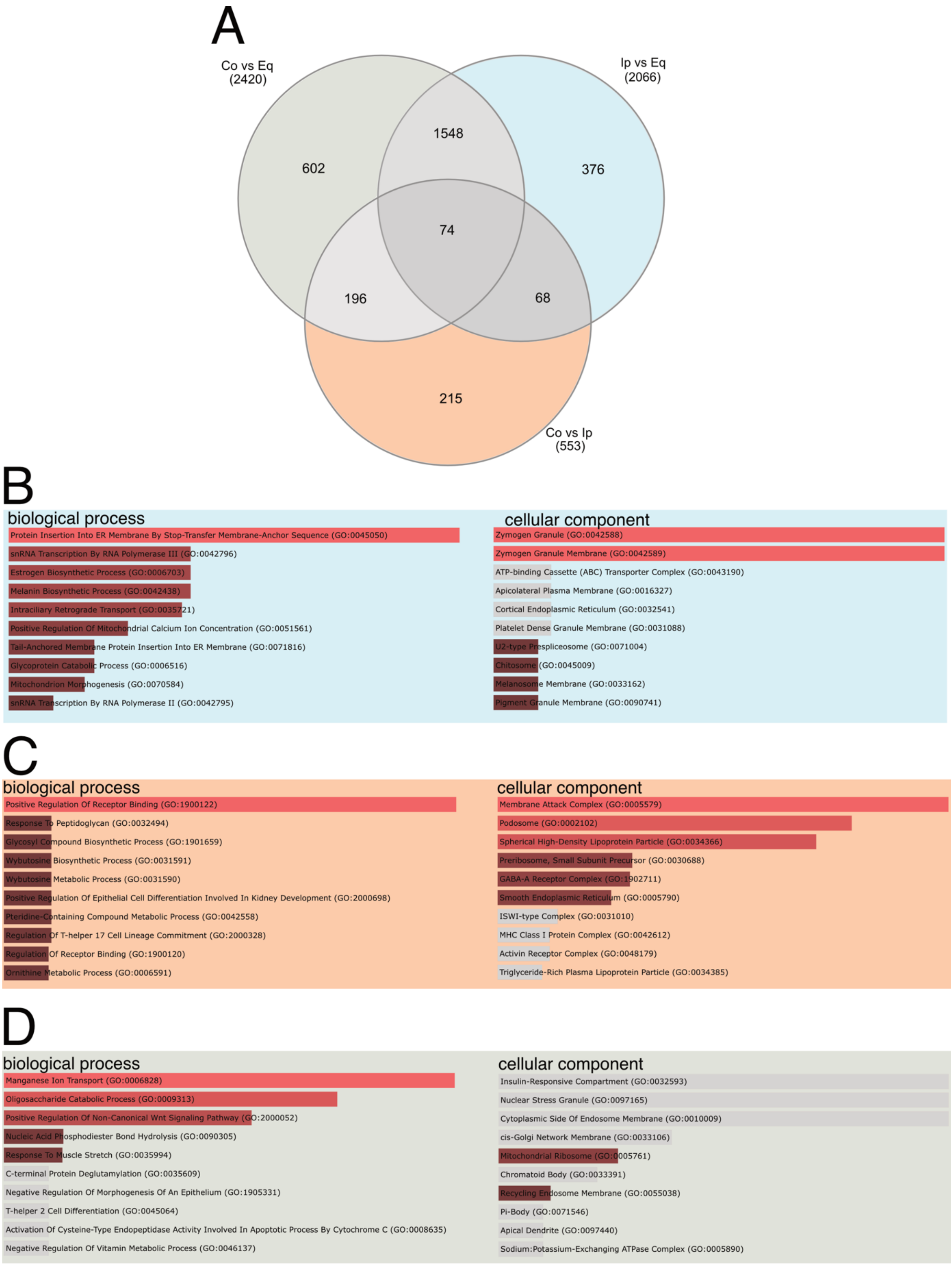

Supplementary figure S2. Further evidence that different fertilization topologies lead to distinct gene expression profiles in 2-cell embryos. (A). Venn diagram representation (rendered with *InteractiVenn*; Heberle *et al.*, 2015) of the differences of gene expression between whole 2-cell embryos of the three ICSI groups**. (B).** Differently expressed genes specific to the comparison between contralateral and equatorial ICSI. **(C).** Differently expressed genes specific to the comparison between ipsilateral and equatorial ICSI. **(D).** Differently expressed genes specific to the comparison between contralateral and ipsilateral ICSI. GO analysis performed with *Enrichr* (Chen *et al.*, 2013).

Supplementary tables

Supplementary table S1. Overview of the number of analyzed genes in this transcriptome study of single blastomeres and blastocysts, with summary of the filtering criteria applied to the RNA-seq data.

	Blastomeres	Blastocysts
Number of Genes in the annotation file (gff3) used for mapping	32285	32285
Number of genes after filtering for expression per cell and mapped reads	21778	17342
Number of non-predicted genes	14194	11370
Number of genes with non-zero TPM median (used for further analyses)	7492	7660

Supplementary table S2. Developmental validation of ICSI zygote viability after 24h immobilization.

Fertilization		Embryo outcome				
		-/-	B/-	B/B	consolidated rate	Sample size
ICSI	contralateral	0.35	0.12	0.54	0.6	233
ICSI	equatorial	0.35	0.17	0.48	0.57	234
ICSI	ipsilateral	0.48	0.14	0.38	0.45	234
NF		0.08	0.18	0.74	0.83	203
		-/-	OG/-	OG/OG	consolidated rate	Sample size
ICSI	contralateral	0.14	0.14	0.71	0.78	14
ICSI	equatorial	0.0	0.4	0.6	0.8	40
ICSI	ipsilateral	0.0	0.29	0.71	0.86	14
NF		0.0	0.33	0.67	0.84	12

NOTE. To confirm viability, the blastomeres of 2-cell embryos were mechanically separated from each other. Rates of dual blastomere survival during bisection were similar across the three groups (contralateral ICSI; 64 ± 12 %, 7 sessions, 233 pairs survived; equatorial ICSI: 60 ± 18 %, 7 sessions, 234 pairs survived; ipsilateral ICSI: 68 ± 16 %, 7 sessions, 234 pairs survived) albeit lower than those of naturally fertilized counterparts (83 ± 16 %, 6 sessions, 203 pairs survived). After bisection, they were followed up through 72 h of development post-bisection i.e. approx. 96 h from ICSI. Given the paired nature of the data, blastocyst (B) formation was scored as B/B (observed in both members of the pair), B/- (observed in one member of the pair), or -/- (observed in neither member). The consolidated rates (1 x rate B/B + ½ x rate B/- + 0 x rate -/-) were different among the ICSI groups (p = 0.0044, chi-square test) and were also lower for ICSI than for NF (p = 8.1E-13, chi-square test). Blastocysts were assayed for further development via the outgrowth (OG) assay. Also in this case, given the paired nature of the data, OG formation was scored as OG/OG (observed in both members of the pair), OG/- (observed in one member of the pair), or -/- (observed in neither member). Total N of pairs examined are provided as 'sample size'. Abbreviations: B, blastocyst; NF, natural fertilization; OG, outgrowth.

Supplementary table S3. TPM values of single blastomeres (55 pairs, 7492 genes)
https://dx.doi.org/10.6084/m9.figshare.24796470

Supplementary table S4. Transcripts of DNA-damage repair genes detected in this study

Gene	Co vs Eq<br>p	Ip vs Eq<br>p	Co vs Ip<br>p
**Nucleotide Excision Repair**			
Ccnh	0.11105	0.16875	0.93310
Cetn2	0.71783	0.17932	0.17267
Ddb1	0.00019	0.00041	0.47284
Ercc1	0.90338	0.39122	0.39537
Ercc4	0.24261	0.52382	0.23911
Ercc5	0.59965	0.20836	0.34217
Gtf2h2	0.32017	0.54512	0.65282
Gtf2h3	0.08057	0.03618	0.78201
Gtf2h4	0.71828	0.50921	0.72637
Gtf2h5	0.00004	0.00046	0.23194
Rad23b	0.16879	0.22462	0.67411
**Base Excision Repair**			
Fen1	0.00667	0.06348	0.19726
Mbd4	0.74096	0.84805	0.84508
Parp1	0.00229	0.01749	0.20167
Parp2	0.00010	0.00168	0.97133
Parp3	0.60008	0.23443	0.18296
Smug1	0.00096	0.00641	0.32139
Ung	0.45066	0.23499	0.66258
**Homologous Recombination**			
Babam1	0.02442	0.05626	0.57614
Bard1	0.01087	0.00476	0.86227
Brca2	0.17287	0.04292	0.41416
Mre11a	0.76537	0.91732	0.58511
Rad51	0.04367	0.01746	0.49461
Rad51b	0.24587	0.53839	0.69471
Rad51d	0.64901	0.35553	0.60314
Rad52	0.64807	0.17112	0.30078
Rad54b	0.08642	0.53775	0.26327
Rad54l	0.00842	0.06753	0.11725
Ssbp1	0.45049	0.14989	0.42604
Sycp3	0.01940	0.10581	0.93718
**Mismatch Repair**			
Pms2	0.00018	0.03262	0.13883
Rfc1	0.84193	0.30454	0.10313
Ssbp1	0.45049	0.14989	0.42604
**Fanconi Anemia Pathway**			
Brca2	0.17287	0.04292	0.41416
Eme2	0.37560	0.85589	0.17606
Ercc1	0.90338	0.39122	0.39537
Ercc4	0.24261	0.52382	0.23911
Faap100	0.29556	0.00211	0.00990
Fan1	0.27080	0.37197	0.80394
Fanca	0.71533	0.19262	0.01603
Fancf	0.09598	0.09128	0.95679
Fancg	0.07420	0.18133	0.51544
Fancl	0.26344	0.05056	0.30300
Fancm	0.13102	0.94248	0.09470
Pms2	0.00018	0.03262	0.13883
Polk	0.00061	0.00442	0.35151
Poln	0.03479	0.23495	0.31991
Rad51	0.04367	0.01746	0.49461
Rev1	0.55570	0.02905	0.04341
Rev3l	0.61622	0.84011	0.69487
Rmi2	0.81770	0.56447	0.68740
Slx1b	0.07243	0.69948	0.02597
Telo2	0.29965	0.55953	0.06859
Usp1	0.34985	0.32243	0.85706
Wdr48	0.07918	0.12434	0.68226
**Non-Homologous End-Joining**			
Dclre1c	0.00087	0.00927	0.16703
Fen1	0.00667	0.06348	0.19726
Lig4	0.00936	0.35635	0.00586
Mre11a	0.76537	0.91732	0.58511
Nhej1	0.00080	0.00233	0.44253
Poll	0.03622	0.01957	0.70589
Polm	0.19546	0.81711	0.05642
**DNA Replication**			
Dna2	0.00815	0.00523	0.91177
Fen1	0.00667	0.06348	0.19726
Mcm5	0.59049	0.46634	0.83904
Mcm6	0.08637	0.07730	0.85054
Mcm7	0.07523	0.17676	0.52183
Pole3	0.14475	0.10416	0.71600
Prim1	0.70162	0.49376	0.13555
Rfc1	0.84193	0.30454	0.10313
Rnaseh2b	0.90055	0.82290	0.87947
Ssbp1	0.45049	0.14989	0.42604

NOTE. Pairwise statistical comparisons of the expression levels (TPM) of DNA repair genes between 2-cell embryos of the ICSI groups (Co, Ip, Eq). Uncorrected P values are provided and the significant ($p<0.05$; t test) values are also highlighted in pink color. Note that no mathematical correction for multiple comparisons was applied. In the first column the members of the family of DNA repair genes are broken down into subfamilies. Abbreviations: Co, contralateral ICSI; Eq, equatorial ICSI; Ip, ipsilateral ICSI; TPM, transcripts per million.

Supplementary table S5. TPM values of intact and twin blastocysts (7660 genes)
https://dx.doi.org/10.6084/m9.figshare.24633843
