# Supplementary figures and images for: Animal and vegetal materials of mouse oocytes segregate at first zygotic cleavage: a simple mechanism that makes the two-cell blastomeres differ reciprocally from the start
Source: Mol Hum Reprod. 2024 Dec 30;31(1):gaae045. doi: 10.1093/molehr/gaae045 (PMC11741683; doi:10.1093/molehr/gaae045)

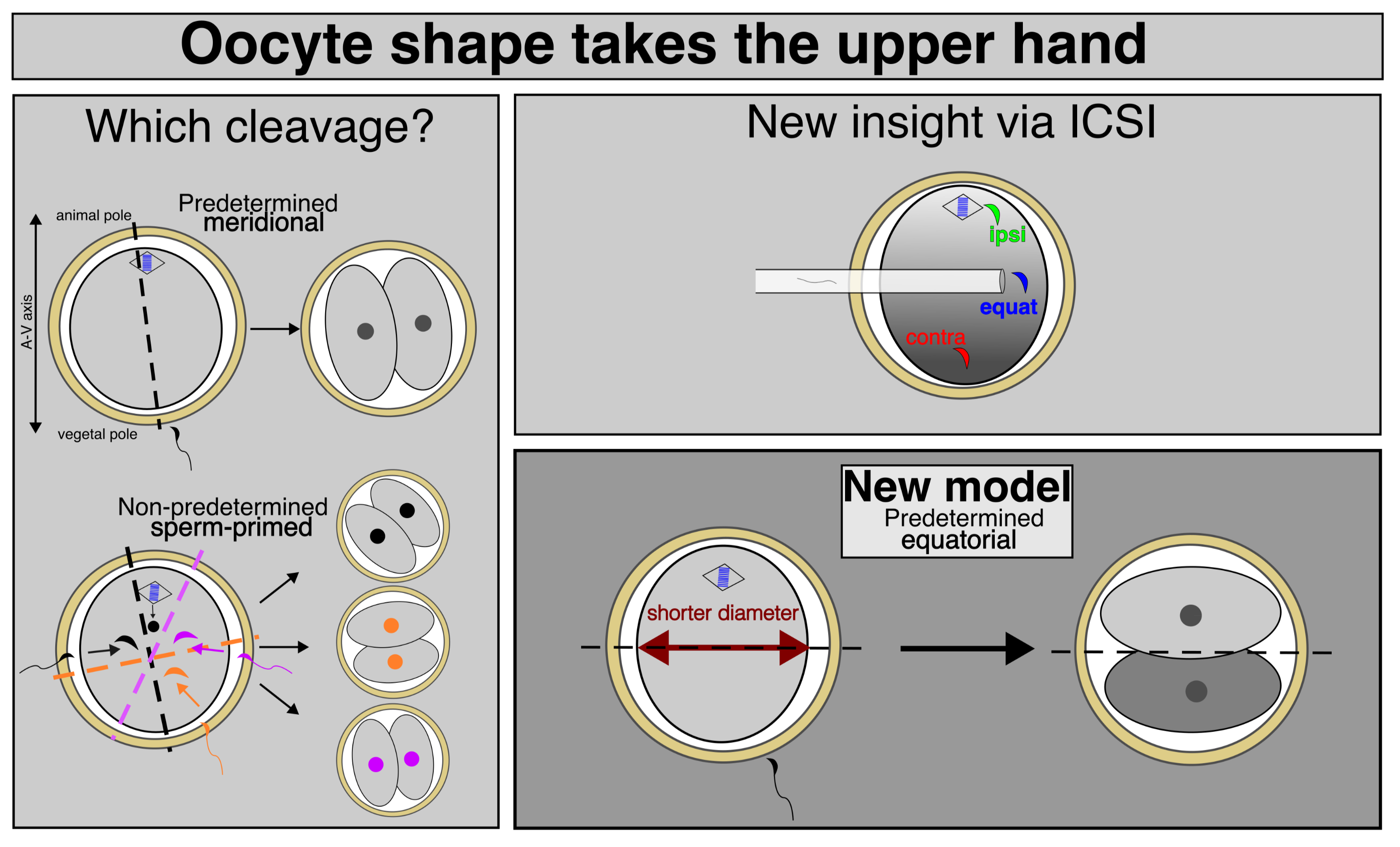

Supplement: gaae045_Supplementary_Data [file gaae045_supplementary_data.zip › 91fa6_Nolte_graphical-abstract.tiff]
